# GFAP hyperpalmitoylation exacerbates astrogliosis and neurodegenerative pathology in PPT1-deficient mice

**DOI:** 10.1073/pnas.2022261118

**Published:** 2021-03-22

**Authors:** Wei Yuan, Liaoxun Lu, Muding Rao, Yang Huang, Chun-e Liu, Shuang Liu, Yue Zhao, Huicong Liu, Jiangli Zhu, Tianzhu Chao, Can Wu, Junyan Ren, Luxian Lv, Wenqiang Li, Shiqian Qi, Yinming Liang, Shijing Yue, Jian Gao, Zhongjian Zhang, Eryan Kong

**Affiliations:** ^a^Institute of Psychiatry and Neuroscience, Xinxiang Medical University, 453000 Xinxiang, China;; ^b^Henan Key Laboratory of Biological Psychiatry, International Joint Research Laboratory for Psychiatry and Neuroscience of Henan, The Second Affiliated Hospital of Xinxiang Medical University, 453000 Xinxiang, China;; ^c^Department of Urology, State Key Laboratory of Biotherapy and Cancer Center, West China Hospital, Sichuan University and National Collaborative Innovation Center, 610041 Chengdu, China;; ^d^School of Medicine, Nankai University, 300071 Tianjin, China;; ^e^Jiangsu Key Laboratory of New Drug Research and Clinical Pharmacy, Xuzhou Medical University, 221000 Xuzhou, China

**Keywords:** GFAP, protein palmitoylation, astrogliosis, neurodegeneration, PPT1

## Abstract

The homeostasis of protein palmitoylation and depalmitoylation is essential for proper physiological functions in various tissues, in particular the central nervous system (CNS). The dysfunction of PPT1 (PPT1-KI, infantile neuronal ceroid lipofuscinosis [INCL] mouse model), which catalyze the depalmitoylation process, results in serious neurodegeneration accompanied by severe astrogliosis in the brain. Endeavoring to determine critical factors that might account for the pathogenesis in CNS by palm-proteomics, glial fibrillary acidic protein (GFAP) was spotted, indicating that GFAP is probably palmitoylated. Questions concerning if GFAP is indeed palmitoylated in vivo and how palmitoylation of GFAP might participate in neural pathology remain unexplored and are waiting to be investigated. Here we show that GFAP is readily palmitoylated in vitro and in vivo; specifically, cysteine-291 is the unique palmitoylated residue in GFAP. Interestingly, it was found that palmitoylated GFAP promotes astrocyte proliferation in vitro. Furthermore, we showed that PPT1 depalmitoylates GFAP, and the level of palmitoylated GFAP is overwhelmingly up-regulated in PPT1-knockin mice, which lead us to speculate that the elevated level of palmitoylated GFAP might accelerate astrocyte proliferation in vivo and ultimately led to astrogliosis in INCL. Indeed, blocking palmitoylation by mutating cysteine-291 into alanine in GFAP attenuate astrogliosis, and remarkably, the concurrent neurodegenerative pathology in PPT1-knockin mice. Together, these findings demonstrate that hyperpalmitoylated GFAP plays critical roles in regulating the pathogenesis of astrogliosis and neurodegeneration in the CNS, and most importantly, pinpointing that cysteine-291 in GFAP might be a valuable pharmaceutical target for treating INCL and other potential neurodegenerative diseases.

## Significance

This study reports that the intermediate filament protein GFAP is modified with protein palmitoylation. Increased GFAP expression and palmitoylation is involved in astrocyte proliferation and astrogliosis. We demonstrate that GFAP palmitoylation is regulated by PPT1, a palmitoylprotein thioesterase linked to a childhood neurodegenerative disorder, infantile neuronal ceroid lipofuscinosis. A palmitoylation-defective mutant of GFAP attenuates astrogliosis and the concurrent pathology in a loss-of-function PPT1 mouse. We conclude that accumulation of palmitoylated GFAP contributes to the pathogenesis of astrogliosis and neurodegeneration, suggesting that targeting the modified cysteine in GFAP may be a potential therapeutic strategy for the treatment of infantile neuronal ceroid lipofuscinosis and other neurodegenerative disorders.

S-palmitoylation can reversibly modify candidate proteins by adding a 16-carbon saturated fatty acid (palmitic acid) on cysteine residues through thioester linkage (1, 2). Giving the hydrophobic nature of palmitic acid, protein palmitoylation is involved in controlling modified proteins in various ways (e.g., protein–protein interaction and cell signaling) (3, 4). The dynamicity of protein palmitoylation is accomplished by the cycling of palmitoylation, catalyzed by DHHC palmitoyl transferases, and depalmitoylation, catalyzed by protein thioesterases (APT1/2, PPT1/2, and Abhd17a) in vivo (5, 6). Remarkably, the disruption of such cycling can cause severe physiological consequences; for example, a common natural mutation in *ppt1* (c.451C > T) results in the dysfunction of PPT1 and thus an early form of neurodegenerative disease infantile neuronal ceroid lipofuscinosis (INCL). Accordingly, PPT1-knockin (KI) mice were generated to mimic this mutation (c.451C > T) for the INCL disease model (7). Of note, one of the typical phenomena in this disease is the gradual loss of neurons associated with increasingly expanded astrogliosis (the activation of astrocytes) in PPT1-KI mice, as also displayed by seizure and shortened longevity in physiological level at later stage of life (6 to 8 mo).

Glial fibrillary acidic protein (GFAP), one of the intermediate filament III proteins, is expressed mainly in astrocytes (astroglia) in the central nervous system (CNS) (8, 9). Upon astrogliosis, GFAP is dramatically up-regulated, accompanied by increased proliferation and cellular size enlargement in astrocytes (10–12). While it seems obvious that GFAP overexpression is closely correlate with the pathogenesis of astrogliosis, the regarding regulatory mechanism is apparently still lacking. Interestingly, in an attempt to identify potential palmitoylated proteins in the CNS by palm-proteomics in our laboratory, GFAP was observed, implying that GFAP is possibly modified with protein palmitoylation. Pursuing the questions respecting whether GFAP is truly palmitoylated in vivo and how palmitoylation might affect the physiological roles of GFAP led us to uncover a pathological mechanism that hyperpalmitoylated GFAP promotes astrogliosis, and blocking of which alleviate astrogliosis and neurodegenerative pathology in INCL mouse model.

## Results

### GFAP Is Palmitoylated at Cysteine 291.

**Fig. 1.**
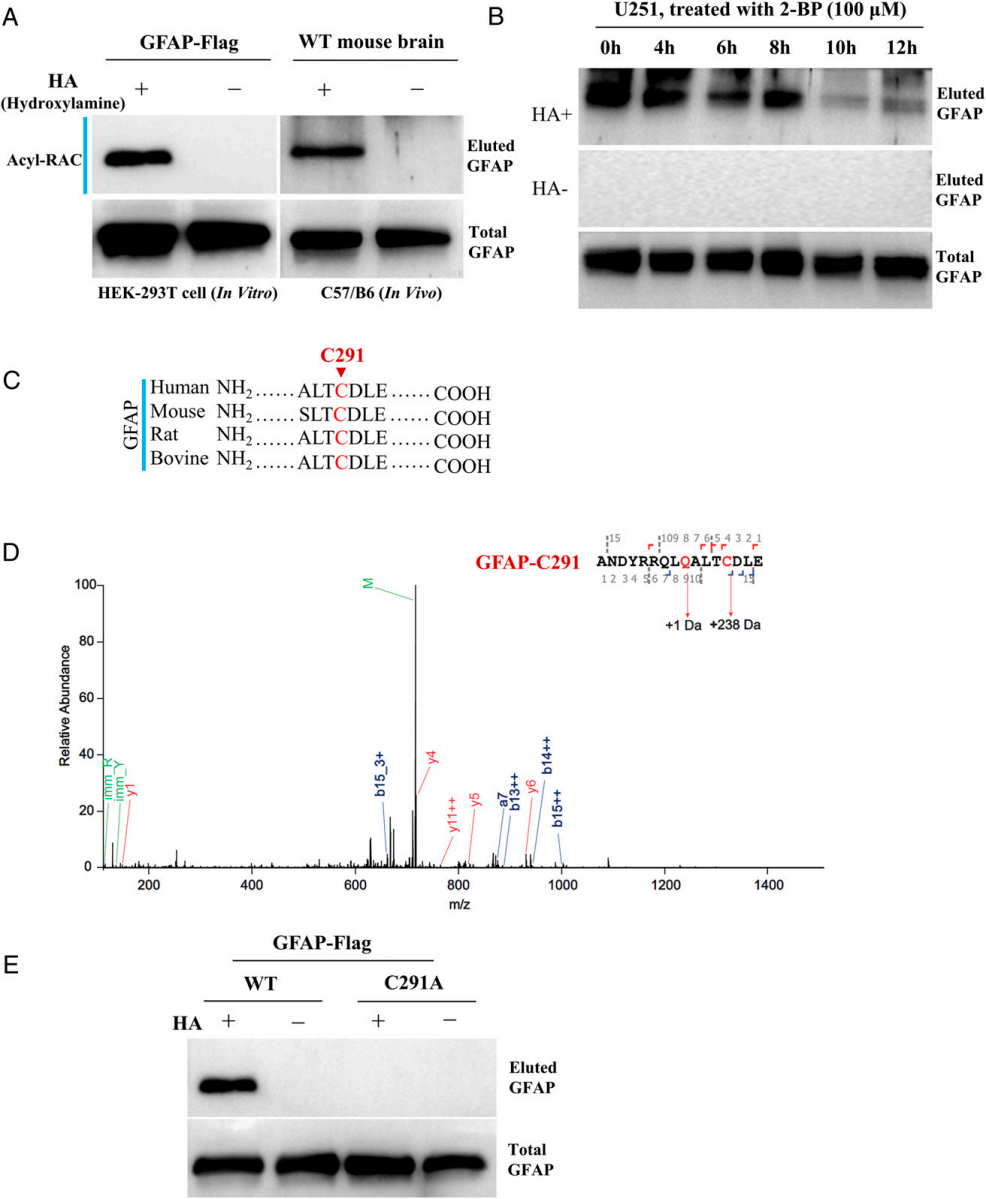
GFAP is palmitoylated at C291. (*A*) GFAP expressed in HEK-293T cells or from mice brain was analyzed for protein palmitoylation by Acyl-RAC assay. HA^+^, with HA, HA^−^, without HA. (*B*) Astroglioma cell line U251 was incubated with 2-BP for different period of time, and then were subjected for evaluating the level of GFAP palmitoylation by Acyl-RAC assay. (*C*) Protein sequence of GFAP from various species were aligned together for analyzing cysteine conservation. (*D*) Purified GFAP was probed by mass-spectrometry, a mass-shift of 238 Da linked to cysteine is a hallmark for palmitoylation. (*E*) WT or point-mutated (C291A, cysteine to alanine mutation) GFAP were expressed in HEK-293T cells and subjected for protein palmitoylation by Acyl-RAC assay.

Initially, GFAP was spotted in a palm-proteomics screening in WT mice brain in our laboratory (*SI Appendix*, Fig. S1*A*), which suggest that GFAP might be palmitoylated. We therefore performed acetyl-resin–assisted capture assay (Acyl-RAC) (*SI Appendix*, Fig. S1*B*) in which palmitoylated-cysteine is converted to nascent thiol group by hydroxylamine (HA) cleavage and thus can be pulled down by thiol-reactive Sepharose resin and eluted for Western blot analysis, to confirm this finding by using either ectopically expressed GFAP in HEK-293T cells or endogenously expressed GFAP in WT mice brain. These results indicate that GFAP is readily palmitoylated both in vitro and in vivo (Fig. 1*A*). To reconfirm, 2-BromoPalmitate (2-BP, a general inhibitor of protein palmitoylation) (3) was incubated with human astroglioma cell line U251, it was found that 2-BP can inhibit the level of palmitoylated GFAP (palm-GFAP) gradually in a manner of incubation time (Fig. 1*B*). Together, these results confirm that GFAP is indeed palmitoylated.

Since S-palmitoylation only occur on cysteine residue (13, 14), we analyzed the protein sequence of GFAP and surprisingly, only one cysteine residue is available. Moreover, the cysteine-291 (C291) is conserved among different species (Fig. 1*C*). It was then easy to conceive that C291 should be the specific palmitoylated residue in GFAP. To directly verify this, GFAP-Flag was expressed, purified, and subjected for evaluation of palmitoylation by mass spectrometry; the data clearly demonstrate that GFAP is modified with palmitate at C291 (with 238 Da of mass alteration) (15, 16) (Fig. 1*D*). Finally, if C291 were mutated into alanine (C291A), then palmitoylation should be entirely diminished in mutant protein GFAP-C291A. Indeed, while GFAP is readily palmitoylated, GFAP-C291A is depleted of palmitoylation (Fig. 1*E*). Hence, it is clear that GFAP is palmitoylated at C291.

### GFAP Palmitoylation Is Involved in Astrocyte Proliferation.

**Fig. 2.**
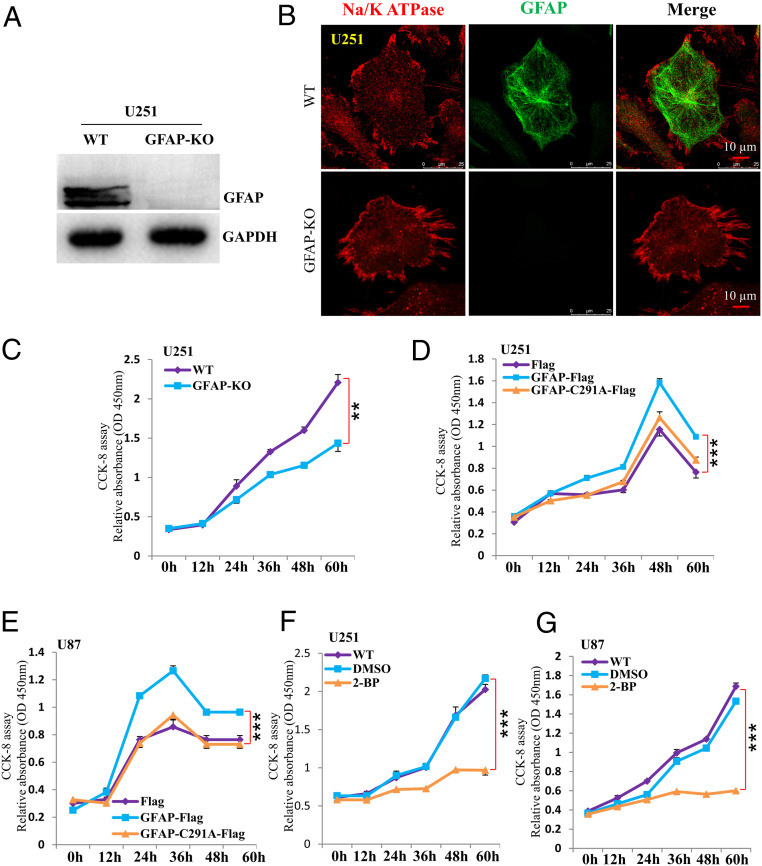
Palmitoylated GFAP is involved in controlling astrocyte proliferation. (*A*) Constructed GFAP-KO cell was examined by Western blot to confirm its loss of GFAP in U251. (*B*) The removal of GFAP does not cause apparent morphological alteration in U251 cell. (*C*) The CCK8 was used to quantify cell proliferation in U251 cells for different genotypes (WT and GFAP-KO). (*D* and *E*) CCK8 was used to quantify cell proliferation in U251 (*D*) or U87 cells (*E*) expressing either empty vector (Flag), GFAP-Flag or GFAP-C291A-Flag construct. (*F* and *G*) CCK8 assay was conducted to quantify cell proliferation in U251 (*F*) or U87 (*G*) as treated with either DMSO, 2-BP, or none (WT). Data are mean ± SEM; *P* values calculated using paired two-sided *t* test. *n* = 3. **P ≤ 0.01, ***P ≤ 0.001.

As astrogliosis occurs in various scenarios of neurodegenerative diseases, the up-regulation of GFAP expression correlate well with the proliferation of astrocytes (10–12), implying that GFAP might boost the expansion of astrocytes population. To assess whether GFAP and its palmitoylation are involved in regulating astrocyte proliferation, we first eliminated GFAP in U251 cell (GFAP-knockout [KO]) (Fig. 2*A* and *SI Appendix*, Fig. S2 *A–C*). While morphologically, there is no obvious defect (e.g., cell size) observed (Fig. 2*B*), the growth of the GFAP-KO cells is dramatically retarded, as confirmed by Cell Counting Kit assay (CCK8) and 5-ethynyl-20-deoxyuridine (EdU) labeling assays (Fig. 2*C* and *SI Appendix*, Fig. S2 *D* and *E*), indicating that GFAP plays an important role in regulating astrocyte proliferation. Next, to specifically evaluate the roles of palmitoylation, GFAP and GFAP-C291A were expressed in both U251 and U87 (human astroglioma) cell lines. The obtained data showed that the expression of GFAP could apparently promote the proliferation of both cell lines, yet the expression of GFAP-C291A literally abolished such effect (Fig. 2 *D* and *E*). To recertify the roles of GFAP palmitoylation in regulating astrocyte proliferation, 2-BP was incubated with both U251 and U87 cells, the results demonstrated that suppressing palmitoylation by 2-BP could greatly reduce the proliferation of both U251 and U87 cells (Fig. 2 *F* and *G*). Together, these data support the conclusion that palmitoylation at C291 is required for GFAP to exert its function in manipulating astrocyte proliferation in vitro.

### GFAP Hyperpalmitoylation Contribute to Accelerated Astrocyte Proliferation in PPT1-KI Mice.

**Fig. 3.**
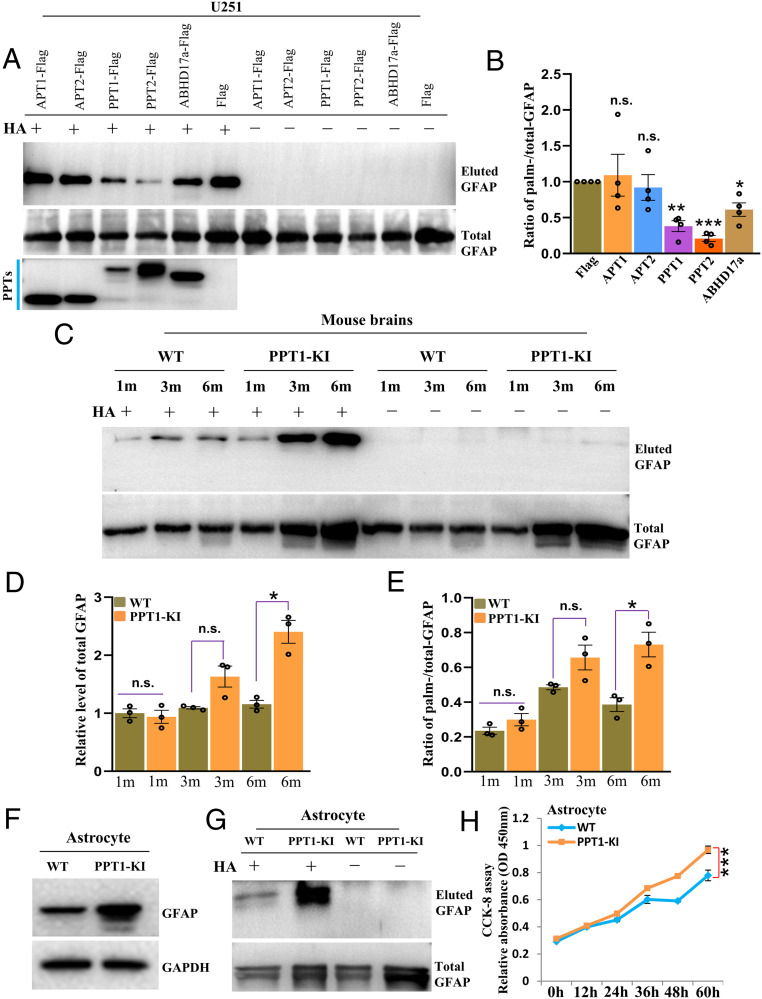
GFAP hyperpalmitoylation stimulates astrocyte proliferation in PPT1-KI mice. (*A* and *B*) All known thioesterases (APT1/2, PPT1/2, and ABHD17a) were coexpressed with GFAP in HEK-293T cells for evaluating their ability to depalmitoylate GFAP in vitro (*A*), and the ratio of palmitoylated-GFAP (*Upper*)/total GFAP (*Lower*) was calculated and quantified (*B*). *P* values calculated using paired two-sided *t* test. *n* = 3. (*C*–*E*) Mice brains of WT and PPT1-KI were used to examine the levels of GFAP and palmitoylated GFAP at different timepoints (1 mo, 3 mo, and 6 mo, *C*); accordingly, the relative level of total GFAP (*D*) and ratio of palmitoylated-GFAP (*Upper*)/total GFAP (*Lower*) was calculated (*E*) and quantified. *P* values calculated using unpaired two-sided *t* test. *n* = 3. (*F* and *G*) Astrocytes isolated from WT and PPT1-KI mice brains were tested for the levels of GFAP (*F*) and GFAP palmitoylation (*G*). (*H*) Isolated astrocytes from WT and PPT1-KI mice brains were cultured and assayed for the rate of cell proliferation by CCK8. *P* value calculated using paired two-sided *t* test. *n* = 3. Data are mean ± SEM; *P ≤ 0.05, **P ≤ 0.01, ***P ≤ 0.001, otherwise not significant (n.s.).

To understand the regulation of GFAP palmitoylation (3), we coexpressed GFAP with all known acyl-protein thioesterases (APTs), APT1/2, PPT1/2 (17), and ABHD17a, which catalyze the action of depalmitoylation. The result showed that the coexpression of PPT1/2 could dramatically reduce the level of palm-GFAP in vitro (Fig. 3*A*), as hinted by the quantification (Fig. 3*B*). To verify this in vivo, we utilized the INCL mouse model, PPT1-KI, where a point mutation is introduced in *ppt1* and result in PPT1 dysfunction, featured by severe astrogliosis and neurodegenerative pathology at later stages (e.g., ≥6 mo) (7). Our data showed that the level of GFAP is significantly up-regulated in PPT1-KI mice at 6 mo as compared to that of the WT mice (Fig. 3 *C*, *Lower*, and Fig. 3*D*), which is in consensus with previous findings (12). Most critically, the absolute and relative level of palm-GFAP (palm-GFAP/total GFAP) is also greatly elevated in PPT1-KI mice at 6 mo (Fig. 3 *C*, *Upper*, and Fig. 3*E*), which prove that PPT1 indeed catalyze the depalmitoylation of GFAP in vivo.

One might question why the level of palm-GFAP is not affected at earlier stages (e.g., 1 M) in PPT1-KI mice, considering that PPT1 catalyzes the depalmitoylation of GFAP (Fig. 3 *C* and *E*); our assumption was that the increased level of palm-GFAP might be diluted by other cell types, considering that astrocyte population is not overwhelmed in PPT1-KI mice at 1 M. We therefore isolated and cultured astrocytes from postnatal day (P)0 mice brain for examining the level of palm-GFAP in WT and PPT1-KI mice. Clearly, the experiments illustrated that the levels of both GFAP and palm-GFAP are elevated in the astrocytes from PPT1-KI as compared to that of the WT mice (Fig. 3 *F* and *G*). Furthermore, we reasoned whether the enhanced level of palm-GFAP would facilitate the proliferation of astrocytes from PPT1-KI mice. Our results, in agreement with above findings (Fig. 2 *D* and *E*), honestly showed that astrocytes isolated from PPT1-KI do grow faster than those from WT mice (Fig. 3*H*). These data hinted that upon the dysfunction of PPT1 in PPT1-KI mice, GFAP as its potential substrate, the level of palm-GFAP is promptly up-regulated, which augment the astrocyte proliferation at the very beginning and possibly contribute to astrogliosis at 6 mo in PPT1-KI mice.

### Blocking C291-Palmitoylation in GFAP Alleviate Astrogliosis in PPT1-KI Mice.

To test the idea that elevated palm-GFAP promote astrocyte proliferation and astrogliosis in PPT1-KI mice, we thought to rescue such phenotype by blocking the palmitoylation in GFAP through a genetic approach, which mutates Cys-291 into alanine in GFAP in PPT1-KI mice (hereafter named PPT1-KI/GFAP-C291A) (*SI Appendix*, Fig. S3). The genotype of GFAP-C291A was also singled out through crossing; all four genotypes presented seem normal at birth in general.

**Fig. 4.**
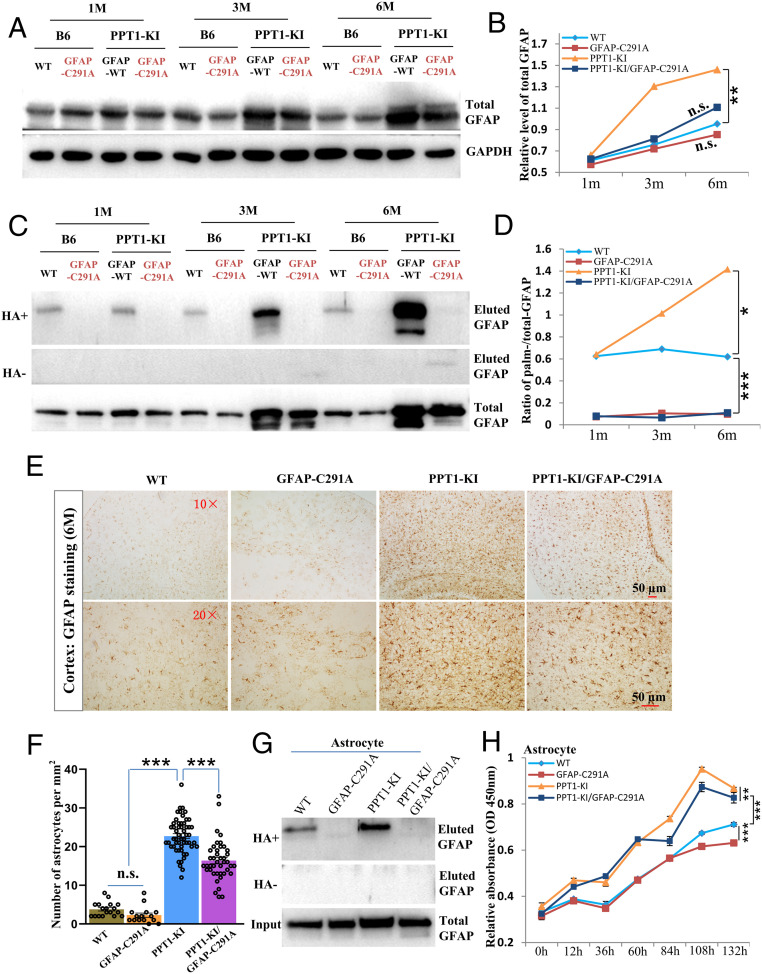
Blocking GFAP palmitoylation down-regulate astrocyte proliferation and alleviate astrogliosis in PPT1-KI mice. (*A*–*D*) Mice brains were collected from different genotypes (WT, GFAP-C291A, PPT1-KI, and PPT1-KI/GFAP-C291A) at various timepoints (1 mo, 3 mo, and 6 mo) and subjected for the evaluation of GFAP (*A*) and GFAP palmitoylation by Acyl-RAC (*C*); correspondingly, the relative level of GFAP expression (*B*) and the ratio of palmitoylated-GFAP (*Upper*)/total GFAP (*Lower*) was calculated (*D*) and quantified. *P* values calculated using paired two-sided *t* test. *n* = 3. (*E* and *F*) Mice brains of different genotypes were paraffin-sectioned and immune-stained with GFAP (*E*) for the quantification of astrocytes in vivo (*F*). Biological replicates are indicated by scattered dots on the bars. *P* values calculated using a one-way ANOVA followed by Dunnett’s test. (*G*) Isolated astrocytes from various genotypes were examined for the levels of GFAP palmitoylation. (*H*) Isolated astrocytes from all genotypes were cultured and assayed for the rate of cell proliferation by CCK8. *P* values calculated using paired two-sided *t* test. *n* = 3. Data are mean ± SEM *P ≤ 0.05, **P ≤ 0.01, ***P ≤ 0.001, otherwise not significant (n.s.).

Both levels of GFAP and palm-GFAP were examined in all genotypes collected at 6 mo; these results repeatedly show that the levels of both GFAP and palm-GFAP are highly up-regulated in PPT1-KI mice as compared to that of the WT and GFAP-C291A mice (Fig. 4 *A*–*D*). As expected, the signal of palm-GFAP is diminished in GFAP-C291A and PPT1-KI/GFAP-C291A mice (Fig. 4*C*). Accordingly, we noticed that the expression of GFAP itself is down-regulated in PPT1-KI/GFAP-C291A as compared to that of the PPT1-KI mice (Fig. 4 *A* and *B*). Yet, the level of GFAP in PPT1-KI/GFAP-C291A mice is nevertheless higher than those of WT and GFAP-C291A mice (Fig. 4 *A* and *B*), which led us to wonder to what extent the accelerated astrocyte proliferation in PPT1-KI is inhibited in PPT1-KI/GFAP-C291A mice.

Mice brains of all genotypes at 6 mo were paraffin-sectioned and subjected for GFAP staining. Not surprisingly, the GFAP^+^ astrocytes are outnumbered in PPT1-KI mice brain (Fig. 4 *E* and *F*). Most interestingly, the number of astrocytes is significantly down-regulated in the brain of PPT1-KI/GFAP-C291A mice as compared to that of the PPT1-KI mice (Fig. 4 *E* and *F*), proving that targeting GFAP palmitoylation indeed alleviate astrogliosis in PPT1-KI mice. Yet, the number of astrocytes remains relatively high in PPT1-KI/GFAP-C291A if compared with WT or GFAP-C291A mice (Fig. 4 *E* and *F*), implying that other unknown factors might also contribute to the astrocyte proliferation (9, 11, 18). Additionally, to reassure that targeting GFAP palmitoylation could manipulate astrocyte proliferation ex vivo, we isolated and cultured astrocytes from all genotypes. These results clearly demonstrated that as GFAP palmitoylation is abolished (Fig. 4*G*), astrocyte proliferation is distinctly down-regulated in GFAP-C291A as compared to WT mice, and in PPT1-KI/GFAP-C291A as compared to PPT1-KI mice (Fig. 4*H*).

### Blocking GFAP Palmitoylation Attenuate Neurodegenerative Pathology in PPT1-KI Mice.

**Fig. 5.**
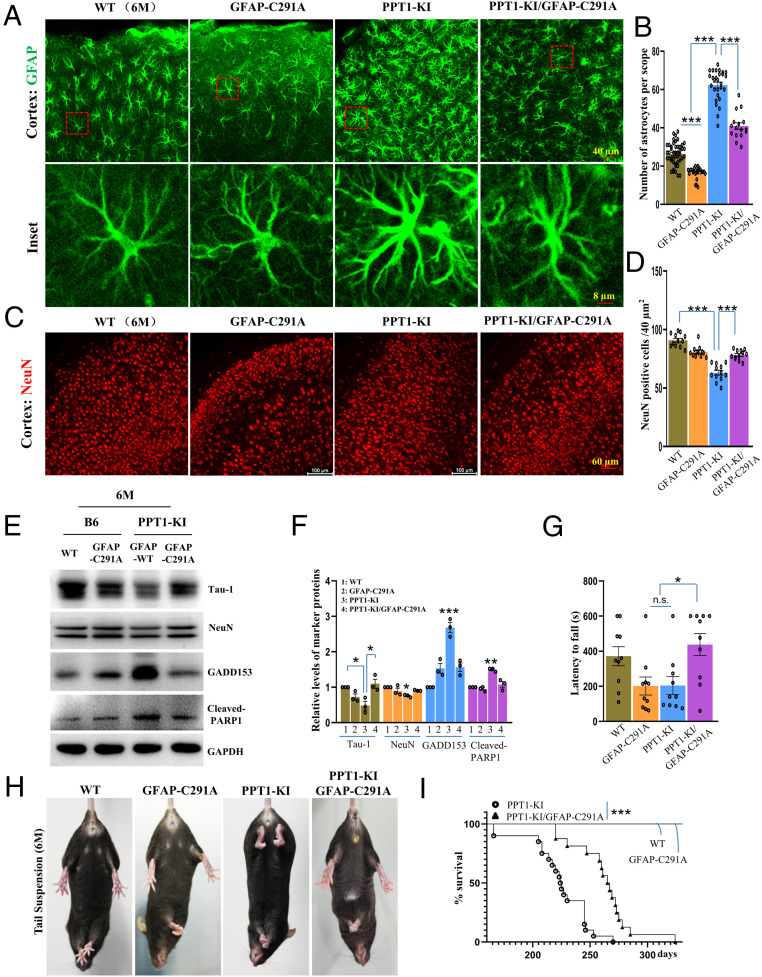
Blocking GFAP palmitoylation partially rescue the neurodegenerative pathology in PPT1-KI mice. (*A*–*D*) Mice brains of all genotypes were processed for transparency with X-clarity and stained for GFAP and NeuN for visualizing astrocytes (*A*) and neurons (*C*) in vivo; the number of astrocytes (*B*) and neurons (*D*) was quantified manually after imaging. (*E* and *F*) Brain lysate from different genotypes were subjected for Western blot (*E*) to evaluate the protein levels of Tau-1 (axonal marker), NeuN (neuronal marker), GADD153 (ER stress marker), cleaved-PARP1 (apoptosis marker), all of which was quantified accordingly (*F*). *n* = 3. (*G*) Rotarod test was carried out in 6-mo-old mice of different genotypes to examine the motor coordination. *n* = 10. (*H*) Tail suspension test was performed for checking seizure behavior in 6-mo-old mice of different genotypes. (*I*) Kaplan–Meier plot comparing lifespans between genotypes. *n* = 18, 14, 20, 16 for wild-type, GFAP-C291A, PPT1-KI, and PPT1-KI/GFAP-C291A, respectively, *P* values calculated using log-rank test. Biological replicates are indicated by scattered dots on the bars. *P* values calculated using a one-way ANOVA followed by Dunnett’s test, except where indicated. Data are mean ± SEM; *P ≤ 0.05, **P ≤ 0.01, ***P ≤ 0.001, otherwise not significant (n.s.).

As known, the event of astrogliosis is often accompanied with the progression of neurodegenerative pathology (e.g., apoptosis, endoplasmic reticulum [ER] stress, and neuronal loss in neurodegenerative diseases) (7, 11, 19, 20). Fascinated by seeing that PPT1-KI/GFAP-C291A could retrieve PPT1-KI mice from astrogliosis (Fig. 4 *E* and *F*), we thought to test whether the associated neurodegenerative pathology could be attenuated by blocking GFAP palmitoylation as well. Mice brains of four genotypes were sliced and processed for transparency by X-CLARITY (*SI Appendix*, Fig. S4) (21, 22), which were then stained with GFAP and NeuN for visualizing the population of astrocytes and neurons in vivo. Our data, again, clearly demonstrated that as astrogliosis is apparently alleviated by cell number (Fig. 5 *A* and *B*), but not morphologically (i.e., size enlargement) (*SI Appendix*, Fig. S5) in PPT1-KI/GFAP-C291A mice, NeuN^+^ neurons are significantly recovered in PPT1-KI/GFAP-C291A as compared to that of the PPT1-KI mice (Fig. 5 *C* and *D*). Intriguingly, the number of astrocytes is also found reduced in GFAP-C291A as compared to that of WT mice (Fig. 5 *A* and *B*), further strengthening the notion that GFAP palmitoylation is essential for astrocyte proliferation both in vitro and in vivo, yet irresponsible for cell size enlargement during astrogliosis.

Biochemically, several marker proteins related to neurodegenerative pathology was measured: that is, Tau-1 (axon), NeuN, GADD153 (ER stress), and cleaved poly (ADP-ribose) polymerase 1 (PARP1) (apoptosis), which were reported obviously altered in 6-mo PPT1-KI mice (7, 19, 20). Consistently, our results verified that severe neurodegenerative pathology occur in PPT1-KI mice, as Tau-1 and NeuN are down-regulated, and concurrrently GADD153 and cleaved-PARP1 are up-regulated in PPT1-KI as compared to that of WT mice (Fig. 5 *E* and *F*). However, this neurological pathology is greatly relieved in PPT1-KI/GFAP-C291A mice in general, as the levels of Tau-1 and NeuN are up-regulated, and the levels of GADD153 and cleaved-PARP1 are down-regulated in PPT1-KI/GFAP-C291A as compared to that of PPT1-KI mice, which is supported by the quantification data (Fig. 5 *E* and *F*).

Physiologically, we thought to test if the recovery of neurological pathology in PPT1-KI/GFAP-C291A mice would improve its neurological functions. A panel of behavior tests was carried out, as the PPT1-KI mice showed a more frequent fall-off from the rotarod test (7, 23), the PPT1-KI/GFAP-C291A mice could sustain much longer on the rod (Fig. 5*G*). Besides, seizure behavior in hitter legs analyzed by tail-suspension, indicative of neurodegeneration, is a typical phenotype for 6-mo-old PPT1-KI mice (7, 23). Notably, this seizure behavior is principally recovered in PPT1-KI/GFAP-C291A mice (Fig. 5*H*). Finally, the life span of all genotypes were examined, persistently, the PPT1-KI mice show an average life span of 6 to 8 mo of age (7, 23), the survival of the PPT1-KI/GFAP-C291A mice prolonged to about 8 to 10 mo, while WT and GFAP-C291A mice display a normal life span (Fig. 5*I*).

## Discussion

**Fig. 6.**
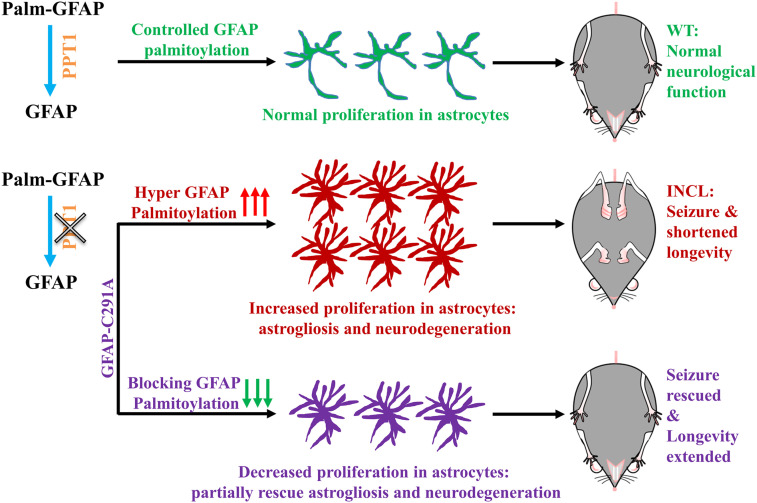
Schematic representation of the pathological mechanism in INCL mice model and a proposed therapeutic strategy. The homeostasis of palmitoylation and depalmitoylation is critical for proper physiological functions in the CNS, which is literally disrupted by the dysfunction of PPT1 in INCL. Specifically, GFAP, as a potential substrate of PPT1, its depalmitoylation process is inhibited by the loss of PPT1 and thus palmitoylated-GFAP is exaggerated in INCL. Importantly, hyperpalmitoylated GFAP promotes astrocyte proliferation and eventually urges the formation of astrogliosis. Blocking GFAP palmitoylation by introducing C291A in GFAP in PPT1-KI mice diminish the pathological burden of astrogliosis and neurodegeneration simultaneously, and therefore rescue seizure and extend longevity in PPT1-KI/GFAP-C291A mice.

Upon the dysfunction of PPT1, the balance of protein palmitoylation and depalmitoylation is disturbed in PPT1-KI mice, especially for those proteins whose depalmitoylation processes are catalyzed by PPT1. Notably, a prominent event develops in the brain of PPT1-KI mice is the association of gradually deteriorating neurodegeneration along with increasingly intensified astrogliosis (7, 24). Whether this tight correlation of astrogliosis and neurodegeneration is a direct consequence of PPT1 dysfunction remain by far not fully understood. Earlier studies in the INCL mouse model suggested that astrogliosis might be triggered by indirect contributors [e.g., oxidative stress (25, 26), ER stress (20, 27), apoptosis (19, 28), and neural inflammation (29)]. Here, we uncovered a pathological mechanism that GFAP-C291 palmitoylation directly control astrocyte proliferation in vitro (Fig. 2 *D*–*G*) and in vivo (Figs. 3*H*, 4*H*, 5 *A* and *B*, and 6). The loss function of PPT1, which depalmitoylates GFAP, results in an elevated level of palm-GFAP in PPT1-KI mice, and therefore accelerates astrocyte proliferation and eventually leads to serious astrogliosis and dampen neurodegenerative pathology in INCL (Fig. 6). Introducing C291A in GFAP in PPT1-KI mice diminishes the pathological burden of astrogliosis, ER stress, apoptosis, and neurodegeneration simultaneously, and accordingly, improves the neurological functions (locomotive activity and seizure) and longevity in PPT1-KI/GFAP-C291A mice (Fig. 6).

Astrogliosis is featured by up-regulated GFAP, morphological hypertrophy (cell size enlargement), and enhanced proliferation in astrocyte (10, 11). If highly expressed GFAP in astrocytes stimulates its morphological hypertrophy and enhanced proliferation is an open question, genetic approaches do point out that the ablation of GFAP and vimentin attenuate astrogliosis (30, 31), hinting that GFAP might contribute to the formation of astrogliosis. Directly, our results demonstrate that GFAP can promote the proliferation of astrocyte in vitro and in vivo; moreover, such effect is dependent on the palmitoylation of C291 in GFAP (Figs. 2 *D*–*G*, 3*H*, 4*H*, and 5 *A* and *B*). Meanwhile, GFAP palmitoylation seems irresponsible for the morphological hypertrophy of astrocyte in PPT1-KI mice, since inhibiting GFAP palmitoylation in vivo (GFAP-C291A) does not rescue the hypertrophic phenotype of astrocyte in PPT1-KI mice (*SI Appendix*, Fig. S5).

Aiming for the treatment of INCL, previous studies reported that applying NtBuHA (23) or *N*-acetylcysteine (12), which could unspecifically break the thioester-linkage connecting palmitate and modified protein in order to ease the burden of hyperpalmitoylation due to PPT1 dysfunction in PPT1-KO mice (12, 32), was proven to be effective. Comparatively, the treatment effect of NtBuHA or NAC in PPT1-KO mice is close to our strategy (blocking C291-palmitoylation) (Fig. 5 *G*–*I*) from the perspective of longevity extension and improvement of neurological functions (12, 23), implicating that hyperpalmitoylated GFAP might be a major target of NtBuHA or NAC as well. Furthermore, in order to correct the overexpressed GFAP and astrogliosis, one study literally deleted GFAP in PPT1-KO mice (33), and surprisingly, the removal of GFAP in whole worsens the neurodegenerative pathology in PPT1-KO mice, hinting that GFAP is involved in more sophisticated physiological functions besides regulating astrogliosis (34–37), and thus the corresponding strategy should be specific if targeting GFAP.

Mechanically, as PPT1 mainly resides in lysosome, one possibility is that GFAP is stably palmitoylated after synthesis until PPT1 depalmitoylates it, targeting for degradation. This assumption is supported by the findings that GFAP is undergoing protein turnover in astrocytes (around 24 h) (*SI Appendix*, Fig. S6*A*) and chloroquine (CHQ, inhibiting lysosome-mediated protein degradation) blocks the turnover of GFAP (*SI Appendix*, Fig. S6*B*). Additional data demonstrated that PPT1, lysosome, and GFAP could colocalize in specific subcellular domains (*SI Appendix*, Fig. S6*B*) and fractions (*SI Appendix*, Fig. S6*C*) in astrocytes, further extending the evidence that PPT1 might depalmitoylate GFAP for degradation. Nevertheless, the exact mechanism of how PPT1 catalyzes GFAP depalmitoylation in vivo remains largely elusive, possibilities are that either palmitoylated-GFAP is translocated into lysosome for depalmitoylation or minimal level of PPT1 is released into cytosol to catalyze this process. Further studies are invited to clarify these points.

Collectively, our study validated that PPT1-dysregulated GFAP hyperpalmitoylation promote astrogliosis and neurodegeneration. Hindering GFAP palmitoylation rescue both astrogliosis and neurodegenerative pathology in PPT1-KI mice and hereby offer palm-C291 in GFAP as a possible pharmaceutical target for curing INCL and other possible neurodegenerative diseases.

## Materials and Methods

### Cell Culture, Transfection, and Treatments.

HEK-293T was obtained from ATCC (CRL-11268) and U251 and U87 were purchased from the Cell Bank of the Chinese Academy of Sciences. HEK-293T, U251, and U87 were grown in DMEM high glucose medium (Gibco) supplemented with 10% fetal bovine serum (Gibco) containing 100 μg/mL streptomycin and 100 U/mL penicillin at 37 °C in a 5% CO_2_ incubator. All cell lines were transfected using Lipofectamine 3000 reagent (Invitrogen) with the reduced serum medium Opti-MEM (Life Technologies) according to the manufacturer’s instructions for 24 h. The follow reagents were used for cells treatments: 2-BP (Sigma-Aldrich, Cat #238422) and DMSO (Sigma-Aldrich, Cat #D8418). All drug experiments were started at 70 to 80% HEK-293T cells confluence in 10-cm dishes. Cells were exposed to fresh DMEM complete medium containing drugs or control DMSO for the indicated time and then washed with PBS three times.

### Plasmids.

The plasmids expressing mouse-origin GFAP-Flag, GFAP-C291A-Flag, APT1-Flag, APT2-Flag, PPT1-Flag, PPT2-Flag, and ABHD17a-Flag were constructed. The cDNA for the mouse GFAP, APT1, APT2, PPT1, PPT2, or ABHD17a was obtained by RT-PCR, which was then subcloned into pUC19 as cDNA donors. Specific primers were synthesized for subcloning individual cDNA into pCMV3-C-Flag for mammalian cell expression by in-fusion cloning method. All constructs were verified by sequence analysis.

### Acyl-RAC.

RAC assay was performed as described previously (14, 38). Briefly, mice brain/cells were collected and washed in cold PBS three times and then lysed in lysis buffer (20 mM Tris pH = 7.5, 150 mM NaCl, 1% Triton X-100) containing protease inhibitor mixture (Roche). Lysate was further processed by sonication and then incubated at 4 °C for 30 min while rotating, which was then cleared by centrifugation at 15,000 × *g* for 10 min at 4 °C, the supernatant was collected. Total protein was quantified with a bicinchononic acid (BCA) assay Kit (Cat #P0009, Beyotime). Next, 1 mg protein lysate was diluted to a concentration of 1 mg/mL in blocking buffer (100 mM Hepes, 1 mM EDTA, 2.5% SDS, 50 mM NEM, pH 7.5) at 50 °C for 60 min with shaking. NEM was then removed by adding three volumes of cold acetone and four sequential 70% acetone precipitations, and pellets were resuspended in 600 μL binding buffer (100 mM Hepes, 1 mM EDTA, 1% SDS, pH 7.5). Samples were equally divided into two parts and added 50 μL prewashed thiopropyl Sepharose 6B (Cat #17-0420-01, GE Healthcare), respectively, one part was added 40 μL 2 M HA pH 7.0 (to cleave thioester bonds) and the other was added 40 μL 2 M NaCl (negative control); 20 μL of each supernatant was taken as the “input.” Cleavage and capture were carried out on a rotator at room temperature for 4 h. Resins were washed five times with binding buffer. For Western blot analysis, elution was performed using 40 μL Leammli loading buffer (2.1% SDS, 66 mM Tris⋅HCl [pH 7.5], 26% [wt/vol] glycerol, 50 mM DTT) on a shaker at 42 °C for 15 min. Supernatants were removed and added 1 μL 5× SDS-PAGE loading buffer, heated to 100 °C for 5 min and analyzed by SDS/PAGE.

### Western Blot Analysis and Antibodies.

Samples were separated in standard SDS-PAGE gels and transferred to Immobilon-P PVDF membrane (pore size 0.2 μM; EMD Millipore). The membrane was then blocked in 5% (wt/vol) skimmed milk in TBS containing 0.1% (vol/vol) Tween-20 (TBST) for 90 min. After blocking the membranes were washed in TBST and incubated with primary antibody for overnight at 4 °C. After washing with TBST, the membranes were incubated with a suitable horseradish peroxidase (HRP)-labeled secondary antibody and signals were detected with an ECL kit (Tanon). The following primary antibodies were used: GFAP (Abcam, Cat#ab7260; 1:10,000 for immunoblot), Tau-1 (MAB3420, Cat #3172074; 1:5,000 for immunoblot), NeuN (Abcam, Cat #ab177487; 1:2,000 for immunoblot), cleaved PARP1 (BD Pharmingen, Cat #BD552596; 1:2,000 for immunoblot), GADD153 (Abcam, Cat #ab11419; 1:1,000 for immunoblot), PPT1 (Abcam, Cat #ab135516; 1:500 for immunoblot), Lamp1 (Proteintech, Cat #21997-1-AP; 1:2,000 for immunoblot), GAPDH (ABclone, Cat #AC033; 1:20,000 for immunoblot). The following secondary antibodies were used for immunoblot: Goat Anti-Mouse IgG (H+L), HRP Conjugate (Protein Biotechnologies, Cat #PMS301; 1:5,000 for immunoblot) and goat anti-rabbit IgG (H+L), HRP conjugate (Protein Biotechnologies, Cat #PMS302; 1:5,000 for immunoblot). GAPDH was served as an internal reference for equal sample loading.

### Protein Purification for Mass Spectrometry.

HEK-293T cells were transfected with pCMV3-GFAP-Flag using Lipofectamine 3000 as described above for 24 h before harvest with lysis buffer (20 mM Tris pH 7.5, 150 mM NaCl, 1% Triton X-100) containing protease inhibitor mixture (Roche). The protein extract was clarified by centrifugation at 15,000 × *g* for 10 min at 4 °C twice. The anti-DYKDDDDK Affinity resin (Sino Biological, Cat. #101274-MM13-RN) was equilibrated in equilibrating buffer (PBS 10 mM pH 7.4) three times and then the precleared lysates were added and incubated at 4 °C with rotation for overnight to pull down the GFAP-Flag. The resin was pelleted by centrifugation and washed in equilibrating buffer (PBS 10 mM pH 7.4) three times and eluted in eluting buffer (100 mM Glycine, 10 mM NaCl, pH 3.0). The resins were removed by centrifugation and the elute supernatants were removed to new tubes. The purified GFAP-Flag was quantified using Coomassie blue staining (Beyotime, Cat#P0017).

### Mass Spectrometric Analysis of Palmitoylation in GFAP.

Purified GFAP-Flag (30 µg) were digested using FASP (39). Briefly, disulfide bonds were broken and blocked using 2 mM Tris (2-carboxyethyl) phosphine (TCEP) and 10 mM iodoacetamide (IAA), then proteins were transferred to 10 K filter, and cleaned sequentially using 8 M urea and 50 mM Tris⋅HCl pH 6.8 at 13,000 × *g*, 20 °C. GluC (P8100S, BioLabs) was added to filter at 1:50 (mass/mass) in 1× reaction buffer and proteins were digested at 37 °C for 16 h. Peptides were collected at 13,000 × g, 20 °C, lyophilized, and stored at −80 °C until use. Raw files were acquired with data dependent acquisition mode using Orbitrap Fusion Lumos (San Jose, Thermo Fisher). Peptide mixture were separated on EasyNano LC1000 system (San Jose, Thermo Fisher) using both C18 (3 µm, 75 µm × 15 cm, homemade) and C4 column (5 µm, 75 µm × 15 cm; Thermofisher) at a flowrate of 600 nL/min. For peptide separation with C18 columns, a 60-min linear gradient was set as follows: 3% B (0.1% FA in ACN)/97% A (0.1% FA in H_2_O) to 8%B in 5 min, 8% B to 20% B in 38 min, 20% B to 30% B in 8 min, 30% B to 90% B in 2 min and stayed 7 min for 90% B. For peptide separation with C4 column, a 60-min linear gradient was set as follows: 3% B (0.1% FA in ACN)/97% A (0.1% FA in H_2_O) to 8%B in 5 min, 8% B to 20% B in 14 min, 20% B to 30% B in 18 min, 30% B to 90% B in 16 min and stayed 7 min for 90% B. For the data acquisition a top 20 scan mode with MS1 scan range *m/z* 350–1550 was used and other parameters were set as below: MS1 and MS2 resolution was set to 120 K and 30 K; AGC for MS1 and MS2 was 4e5 and 1e5; isolation window was 1.6 Th, dynamic exclusion time was 15 s. To better identify modified amino acid sites, each precursor ion was fragmented with both HCD and EThcD. Collision energy of HCD was set to 32, and for EThcD collision energy was 25 and ETD reaction time was set automatically according to *m/z* and ion charge state of each precursor. Raw files were searched against target protein sequence using Byonic v2.16.11 (Protein Metrics). Searching parameter was set as follows: enzyme of GluC (semi) with maximum number of three missed cleavages; precursor and fragment ion mass tolerance was set to 20 ppm and 50 ppm; variable modification was set to oxidation of M, deamidation of N, Q, carbamidomethylation of C, acetylation of protein N-term, palmitoylation of C, Y, S, T, W. An automatic score cut was used to remove low score peptides. A manual check was applied to further filter high confident palmitoylated cysteine sites. Modified peptides only with continuous b and y product ions can be considered as a high confident modified site.

### Immunofluorescence Staining and Imaging.

Cells were plated onto poly-d-lysine–coated coverslips and transfected as described with indicated plasmids. At 24 h posttransfection, the cells were fixed with 4% (wt/vol) paraformaldehyde (Electron Microscopy Sciences, Cat#15710) and perforated with 0.1% (vol/vol) Triton X-100 in PBS, after blocking in 3% BSA in PBS. Cells were stained with primary antibody GFAP (Abcam, Cat #ab7260, 1:1,000), Na/K ATPase (Abcam, Cat #ab76020; 1:300) and then incubated with secondary antibodies anti-rabbit IgG (H+L) Alexa Fluor Plus 488 conjugated (Invitrogen, Cat #A11070; 1:1,000) and phalloidin-conjugated Alexa Fluor 568 (Invitrogen, Cat #A12380; 1:500), washed, and mounted onto slides with Dapi-Fluoromount-G (Electron Microscopy Sciences, Cat #17984-24). The fluorescence images were examined with a stimulated emission depletion microscopy (Leica TCS SP8 STED).

### Deleting GFAP in U251 Cell.

The online tool (crispor.tefor.net) was used to design single-guide RNA (sgRNA) against the third exon in GFAP (Gene ID: ENST00000588735.2). For cloning purpose, a BbsI restriction site was added. The sgRNA were synthesized by Shanghai Bioligo Biotechnology. The Px458 vector was digested by BbsI (New England Biolabs #R3539), annealed with gRNA and transferred to DH5α competent cells (Beijing protein Biotechnology) for transformation. The positive clones (ampicillin‐resistant) were screened and sequenced by Wuhan Genecreate Bioengineering. The recombinant plasmid was extracted by Tiangen kit (Tiangen Biotechnology) for maximum preparation. Prepared U251 cells were transfected with two pX458 vectors by Lipofectamine 3000 Transfection Reagent (Invitrogen). After 48 h, single fluorescence cell was sorted to 96‐well plate by FACS (BD Biosciences). After about 2 wk, single-cell colonies were obtained. Respective DNA was extracted from these cells and processed with PCR amplification and sequencing for positive clone. The PCR conditions were as follows: 35 cycles including 94 °C for 2 min, 94 °C for 30 s, 55 °C for 30 s, and 72 °C for 30 s, followed by a final extension at 72 °C for 2 min.

### CCK8 Assay.

Cells cultured in a 10-cm dish were transfected with Lipofectamine 3000 reagent (Invitrogen) mixed with equal amounts of plasmids (e.g., Flag, GFAP-Flag, and GFAP-C291A-Flag) for 24 h, then all cells were placed in a 96-well plate and seeded with a density of 1 × 10^4^ cells per well. The proliferation rate of treated cells was determined by CCK8 (Kumamoto) by measuring the Absorbance at 450 nm using a Biotek reader (Infinite M200 Pro, Tecan). All treatments were replicated for at least three times.

### EdU Assay.

Cells were cultured in 96-well plates at 1 × 104 cells per well at 37 °C in a 5% CO_2_ incubator. For labeling, cells were firstly incubated with 50 μM of EdU (Ribobio) for 2 h at 37 °C, then were washed twice with PBS and fixed with 4% formaldehyde for 30 min, followed by the treatment of 0.5% Triton X-100 for 10 min at room temperature. Finally, after washing twice with PBS, each well of cells was reacted with100 μL 1× Apollo reaction mixture for 30 min. The DNA contents were stained with 100 μL of 1× Hoechst 33342 for 30 min. For evaluation, corresponding samples were imaged with fluorescent microscope for the quantification of newly proliferated cells and total cells.

### Cycloheximide or CHQ Treatment.

Cycloheximide (CHX; 100 µM; Sigma, Cat #C7698) or CHQ (50 µM; Sigma, Cat #C6628) was incubated with cultured cells for treatment. Transfected U251 cells were treated with CHX or CHQ for various period of time (e.g., 0 to 24 h), then the treated cells were harvested and processed for Western blotting analysis.

### OptiPrep Gradient Fractionation.

OptiPrep gradient fractionation was performed as described previously (40). Briefly, seven gradient iodixanol solutions (5%, 10%, 15%, 20%, 25%, 30%, and 35%) were prepared by mixing dilution buffer with 40% (wt/vol) iodixanol. Then, the gradient solution was slowly added to the centrifuge tube in descending order of concentration. Cultured cells were rinsed with PBS (twice) and Solution A (0.25 M sucrose, 140 mM NaCl, 20 mM Tris⋅HCl, pH 8.0) once. Solution B (0.25 M sucrose, 140 mM NaCl, 1 mM EDTA, 20 mM Tris⋅HCl, pH 8.0) was added to collect the cells, which were then treated by freeze/thaw cycle three times in liquid nitrogen. Cell nuclei were pelleted by centrifugation at 800 × *g*, 4 °C for 5 min, the postnuclear supernatant was retained. The postnuclear supernatant sample was placed on top of the iodixanol gradient and centrifuged at 100,000 × g for 18 h without acceleration/deacceleration (Thermo Scientific Sorvall WX+ series, swinging bucket Rotor TH-641). After centrifugation, 14 fractions were collected for Western blotting analysis.

### Generation of GFAP Point Mutation Mice.

C57BL/6 (B6) mice were purchased from Beijing Vital River Laboratory Animal Technology and PPT1-KI mice were kindly provided as a gift by Anil B. Mukherjee, National Institutes of Health, Bethesda, MD (7). Fertilized B6 eggs (in vitro fertilization with sperm of PPT1-KI mice) were collected and injected via a microinjection system as described previously with little modification (41). In brief, Cas9 mRNA and sgRNA were produced by using in-vitro transcript (IVT) kits, the single-stranded oligonucleotide donor DNA (ssODN) was synthesized by the Bioligo company, all these components were mixed and microinjected into the cytoplasm of fertilized eggs. Injected eggs were cultured to two-cell stage and then transferred into ICR foster mice. Twenty days later, F0 mice were born and genomic DNA was isolated. The primers: F-GAG​AGA​GCG​CTG​ACT‐​GAG​GT and R-CGCTCTAGGGACTCGTTCTG were used for genotyping. All animal procedures were performed according to guidelines approved by the committee on animal care at Xinxiang Medical University.

### Primary Astrocyte Culture.

Primary astrocytes were prepared from newborn pups (P0) of corresponding mice brains (WT, GFAP-C291A, PPT1-KI, and GFAP-C291A/PPT1-KI mice). Briefly, in order to collect the cerebral cortex, meninges and other parts of the brain were removed. Then, the cerebral cortex was washed three times with 1× HBSS (Thermo Fisher Scientific) and digested by trypsin (0.05%) for 5 to 10 min at 37 °C. Afterward, cells were briefly centrifuged (500 rcf for 5 min), suspended in 10 mL of glial medium (ice-cold, DMEM with 10% FBS and N2-supplement; Gibco, 17502-048), seeded onto collagen-coated plates, and cultured in a 5% CO_2_ humidified incubator at 37 °C. The medium was replaced with fresh medium every 24 h and cultured till 90% confluence. Finally, astrocyte cultures were deprived of oligodendrocytes and microglia by shaking (180 rpm) overnight at 37 °C.

### Immunohistochemistry and Astrocyte Counting.

Mice was anesthetized, perfused and fixed with cold PBS containing 4% paraformaldehyde and then processed for paraffin-sectioning. Briefly, the paraffin sections were dehydrated, sealed in 3% BSA, and incubated with primary antibody (GFAP, Abcam, ab7260; 1:2,500) overnight at 4 °C. The slices were then washed and incubated with secondary antibody of the corresponding species at room temperature for 1 h. DAB staining was applied after cleaning. Images were taken under the same settings of parameters with bright-light Nikon microscope using 20× objectives.

Cell counts were finished majorly within the cortical portion of the brain, 10 images from 3 different brains were collected for all quantifications.

### Transparency and Staining of Mice Brain Sections.

Mice was anesthetized, perfused, and brain tissue was transferred and fixed externally with cold PBS containing 4% paraformaldehyde for no more than 24 h. Afterward, the X-Clarity Tissue Clearing System (Logos Biosystems) was used to conduct transparent treatment of mice brain tissues: Hydrogel was incubated with corresponding samples for 24 h, followed by polymerization for 3 h and electrophoresis for 12 h for removing lipids, and finally tissue was turned almost transparent. For immunofluorescence staining, primary antibodies—that is, GFAP (Abcam, ab7260; 1:100); NeuN (Abcam, ab177487; 1:2,000)—were used. Next, 1% PBST (37 °C, away from light) was added to remove unspecific binding for 24 h. Alexa Fluor-488 (Invitrogen, Cat #A11070; 1:1,000) and Alexa Fluor-594 (Invitrogen, Cat #A11072; 1:1,000) were used as corresponding secondary antibodies where suitable. Finally, 1% PBST and distilled water was sequentially added for clearing before mounting. Images were observed under STED microscopy (Leica TCS SP8 STED). XY or XYZ scan was performed accordingly under same settings of various parameters (e.g., exposure time and magnification). Cell counts were finished majorly within the cortical portion of the brain, 10 images from 3 different brains were collected for all quantifications.

### Behavior Tests for Motor Coordination and Seizure Behavior.

Motor coordination of the WT, GFAP-C291A, PPT1-KI, and PPT1-KI/GFAP-C291A mice was assessed using a rotarod (YLS-4C Rotary Rod Fatigue Tester, Equipment Station of Shandong Academy of Medical Sciences) performance test at a fixed speed of 30 rpm while rotating in a single direction. Animals were trained twice daily at 60-s intervals for 3 consecutive days. They were allowed to rest for 60 s between the two trials. Rotarod experiments were performed for 600 s on day 4 and the amount of time a mouse was on the rotarod before falling from the rotating rod was recorded. Tail suspension test was applied to evaluate the seizure behavior in the hitter legs of different genotypes. Behavioral analyses were carried out by an experimenter blinded to the experimental groups.

### Longevity.

Different genotypes of mice (*n* = 15 to 20 mice per genotype) were used to assess lifespan. Mice from each group were allowed to age, unmanipulated (the same living environment). Under the above conditions, death or a predetermined dying state (inability to drink or eat properly) marks the end of life.

### Statistical Analysis.

Basic descriptive data are presented as means SEs of means. For statistical analyses of differences between two groups, paired or unpaired two-tailed Student’s *t* tests were used where appropriate. For experiments involving more than two groups, one-way ANOVA analysis was carried out. Post hoc pairwise comparisons, with Dunnett’s correction for multiple comparisons, were conducted where appropriate. An α-level of 0.05 was adopted in all instances. All analyses were carried out using SPSS 19 professional software (IBM). Graphs were created using GraphPad Prism software for Windows, v5.

## Supplementary Material

Supplementary File

## Data Availability

All study data are included in the article and *SI Appendix*.
