# Supplementary material for: GFAP hyperpalmitoylation exacerbates astrogliosis and neurodegenerative pathology in PPT1-deficient mice
Source: Proc Natl Acad Sci U S A. 2021 Mar 22;118(13):e2022261118. doi: 10.1073/pnas.2022261118 (PMC8020761; doi:10.1073/pnas.2022261118)

Supplementary information titles and legends

Fig. S1. Technical procedures for Palm-proteomics and Acyl-RAC assay. A, Schematic procedures for palm-proteomics, briefly, WT brain lysate was purified by ABE assay for total palmitoylated proteins, and further identified by LC-MS/MS for protein ID. **B**, Schematic procedures for Acyl-RAC assay, similar to ABE assay, after blocking free thiol-group with NEM (N-ethylmaleimide), the thioester linkage between –SH group provided by cysteine and palmitate is cleaved by hydroxylamine (HA, NH2OH); then the freed thiol-group is capable of binding with commercialized beads (biotin beads or SX-S-beads) through disulfide bond. Last, the captured proteins were eluted with β-mecaptoethanol for further analysis.

Fig. S2. Deleting GFAP in U251 inhibits cellular proliferation. A, Targeting scheme of the truncation in the third exon of human GFAP. The deletion mutation was introduced into U251 cell using Crispr/Cas9 with two gRNA. Red box indicates exons, gray box indicates UTRs. Black lines indicate two cut sites in the third exon of human GFAP; **B**, The sequences of WT genomic DNA and mutation DNA. 53bp were deleted from the mutation DNA; **C**, Representative sequencing result of GFAP deletion mutation in U251 cell. The reading frame was changed according to the deleted truncation. **D-E**, U251 cells were processed with EdU assay for the evaluation (**D**) and quantification (**E**) of newly proliferated cells, EdU labels new born cells while Hoechst labels all nuclei. Biological replicates are indicated by scattered dots on the bars. p-value was calculated using unpaired t-test, two-sided. Data are mean ± s.e.m. *$p \leq 0.05$, **$p \leq 0.01$, ***$p \leq 0.001$.

Fig. S3. Generation of GFAP-C291A point mutation mouse. A, Targeting scheme of the point mutation (C291A) in the fifth exon of mouse GFAP, the point mutation was introduced into mouse fertilized eggs using Crispr/Cas9 with the HDR donor DNA. Blue box indicates exons, gray box indicates UTRs. Red star indicates a CTGC→TGCA mutation in the fifth exon of mouse Gfap; **B**, The sequences of WT genomic DNA and donor DNA. The C to t (yellow) mutation was designed to destroyed

the PAM sequence without change the amino acid sequence, TGC to gca (green) was designed to change the amino acid Cys to Ala; **C**, Representative sequencing result of GFAP-C291A point mutation mice. The mutation TGC to gca was underlined and the changed amino acid Cys to Ala was in red.

Fig. S4. Technical process for tissue transparency by X-CLARITY. A, Schematic procedures for removing lipid from brain tissue or brain slices, briefly, PFA fixed mouse brain was incubated with hydrogel monomers for hybridization, electrophoresis was applied to remove lipids from brain tissue for tissue transparency. Cleared tissue was then incubated with corresponding antibodies (1st and 2rd) for immunostaining and subsequent imaging.

Fig. S5. GFAP palmitoylation might not relate to the morphological enlargement of astrocyte in PPT1-KI mice. A, 3D images of individual astrocytes from different genotypes were extracted and projected on XY plane for visualizing the morphological details. b-d, Cell size in area (**B**), number of branches (**C**) and width of major branches (**D**) were measured and quantified manually. n=6. Biological replicates are indicated by scattered dots on the bars. p-values calculated using a one-way ANOVA followed by Dunnett's test. Data are mean ± s.e.m. $^{*}p \leq 0.05$, $^{**}p \leq 0.01$, $^{***}p \leq 0.001$, otherwise not significant (n.s.).

Fig. S6. PPT1 catalyzes GFAP depalmitoylation for possible protein degradation. A-B, Cultured U251 cells expressing GFAP-Flag or GFAP-C291A-Flag were treated with cycloheximide (CHX, 100 μM) and/or Chloroquine (CHQ, 50μM) for the evaluation of protein half-life at various timepoints. C, Cultured U251 cells were transfected with RFP-Lamp1 and PPT1-Flag for 24h, fixed with PFA and stained with Flag and GFAP antibodies for immunofluorescence and colocalization analysis. D, Astrocytes from wild-type mouse brain were isolated and prepared for OptiPrep^{TM} gradient fractionation, in total, 14 fractions were collected for examining the distributions and levels of GFAP, PPT1 and Lamp1 respectively. E, Cultured

hippocampal neurons and astrocytes from wild-type mouse brain were harvested and analyzed for the protein levels of GFAP and PPT1. F, WT brain slices (X-CLARITY processed) were stained with GFAP and imaged for GFAP positive cells *in vivo*.

Fig. S7. Uncropped blot of Fig. 1A, Fig. 1B and Fig. 1E.

Fig. S8. Uncropped blot of Fig. 3A, Fig. 3C, Fig. 3F and Fig. 3G.

Fig. S9. Uncropped blot of Fig. 4A and Fig. 4C.

Fig. S10. Uncropped blot of Fig. 5E.

Fig. S1

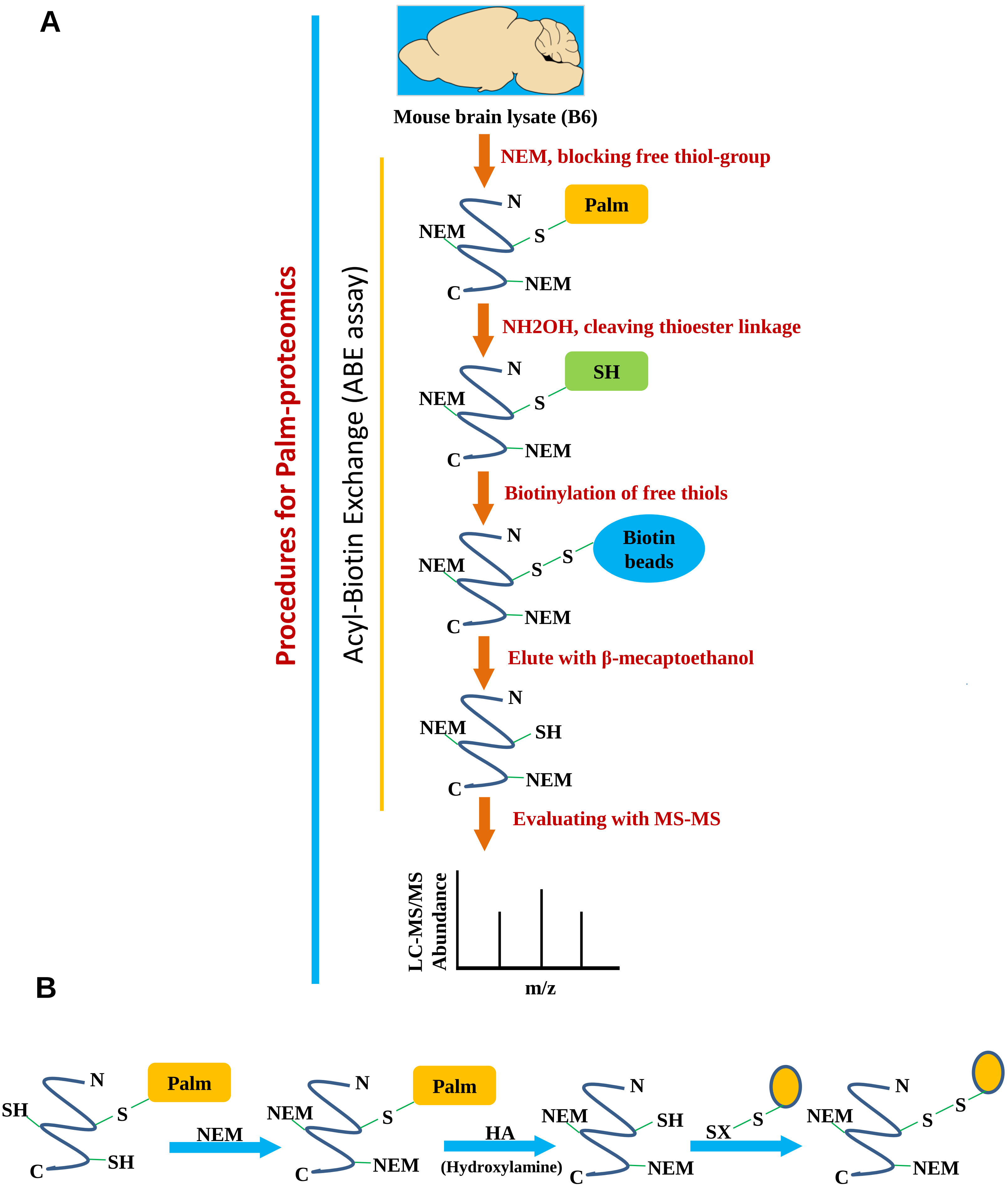
A
Procedures for Palm-proteomics
Acyl-Biotin Exchange (ABE assay)
Mouse brain lysate (B6)
NEM, blocking free thiol-group
N
Palm
NEM
S
NEM
C
NH2OH, cleaving thioester linkage
SH
Biotinylation of free thiols
Biotin beads
Elute with β-mecaptoethanol
Evaluating with MS-MS
LC-MS/MS Abundance
m/z
B
SH
NEM
HA
(Hydroxylamine)
SX

Fig. S2

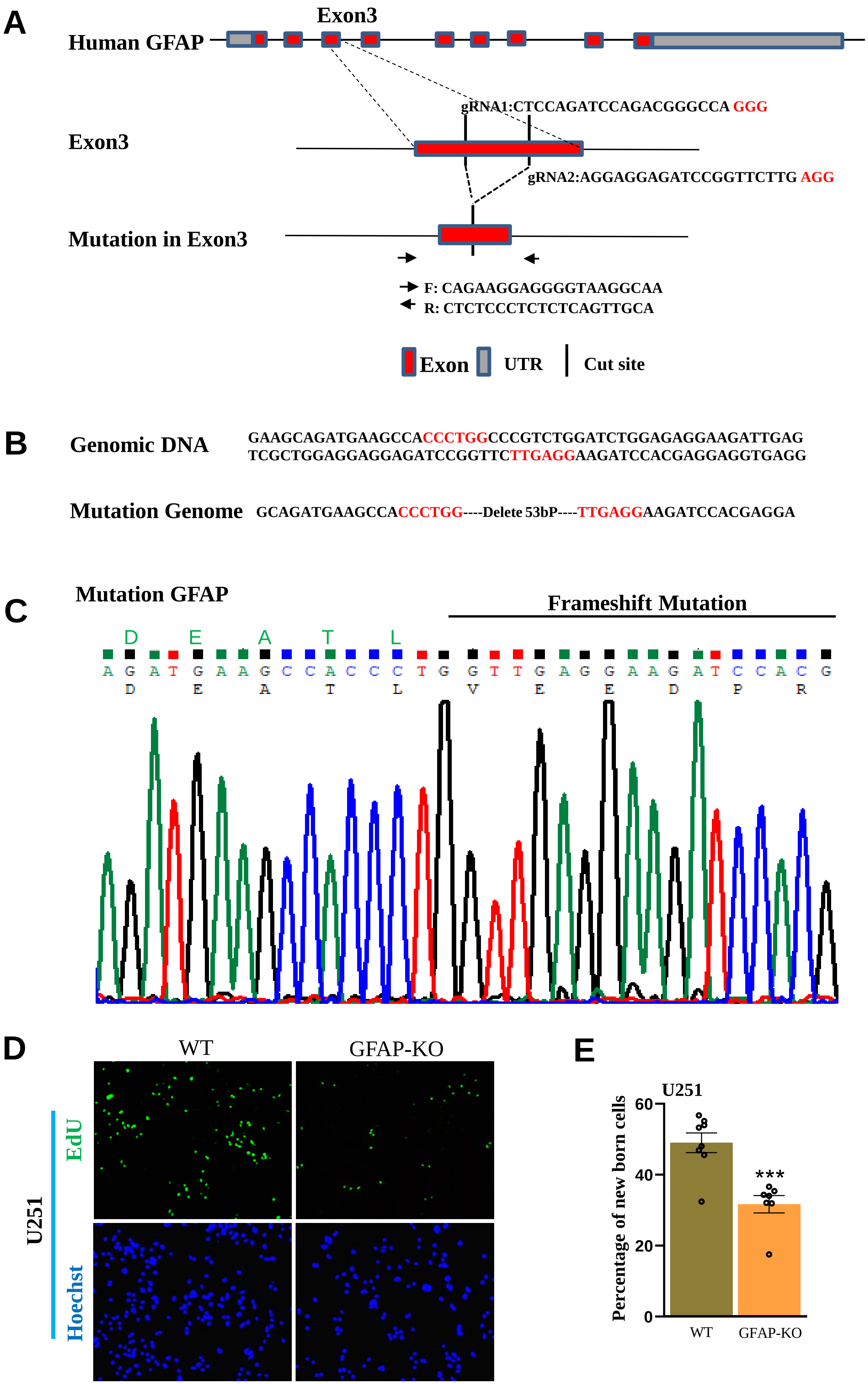
A
Exon3
Human GFAP
gRNA1:CTCCAGATCCAGACGGGCCA GGG
Exon3
gRNA2:AGGAGGAGATCCGGTTCTTG AGG
Mutation in Exon3
F: CAGAAGGAGGGGTAAGGCAA
R: CTCTCCCTCTCTCAGTTGCA
Exon
UTR
Cut site
B
Genomic DNA
GAAGCAGATGAAGCCACCCTGGCCCGTCTGGATCTGGAGAGGAAGATTGAG
TCGCTGGAGGAGGAGATCCGGTTCTTGAGGAAGATCCACGAGGAGGTGAGG
Mutation Genome
GCAGATGAAGCCACCCTGG----Delete 53bP----TTGAGGAAGATCCACGAGGA
C
Mutation GFAP
Frameshift Mutation
D E A T L
A G A T G A A G C C A C C C T G G T T G A G G A A G A T C C A C G
D E A T L V E E D P R
D
WT
GFAP-KO
U251
EdU
Hoechst
E
U251
Percentage of new born cells
60
40
20
0
***
WT
GFAP-KO

Fig. S3

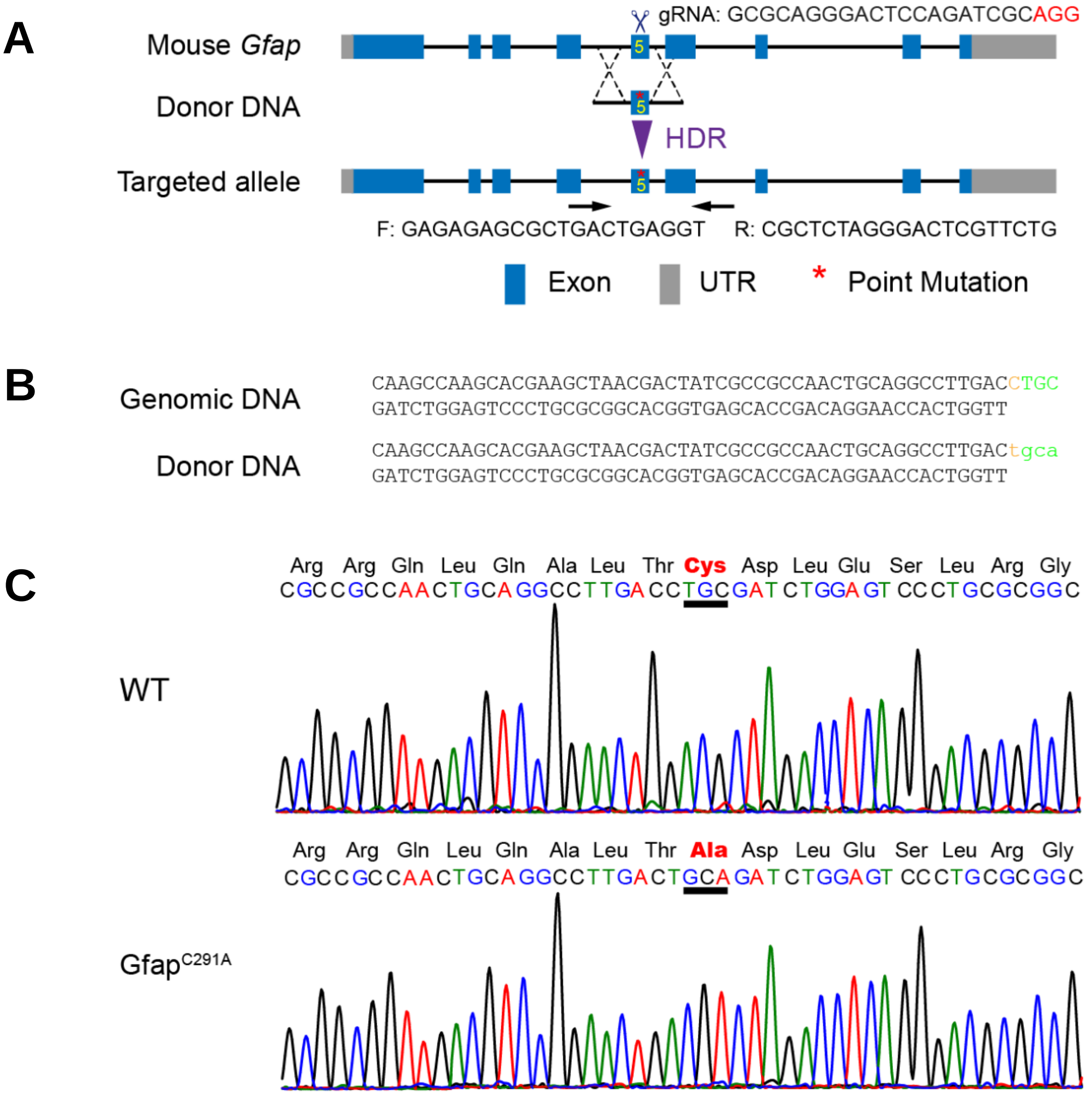
A
Mouse Gfap
gRNA: GCGCAGGGACTCCAGATCGCAGG
5
Donor DNA
5
HDR
Targeted allele
5
F: GAGAGAGCGCTGACTGAGGT
R: CGCTCTAGGGACTCGTTCTG
Exon
UTR
* Point Mutation
B
Genomic DNA
CAAGCCAAGCACGAAGCTAACGACTATCGCCGCCAACTGCAGGCCTTGACCTGC
GATCTGGAGTCCCTGCGCGGCACGGTGAGCACCGACAGGAACCACTGGTT
Donor DNA
CAAGCCAAGCACGAAGCTAACGACTATCGCCGCCAACTGCAGGCCTTGACtgca
GATCTGGAGTCCCTGCGCGGCACGGTGAGCACCGACAGGAACCACTGGTT
C
Arg Arg Gln Leu Gln Ala Leu Thr Cys Asp Leu Glu Ser Leu Arg Gly
CGCCGCCAACTGCAGGCCTTGACCTGCGATCTGGAGTCCCTGCGCGGC
WT
Arg Arg Gln Leu Gln Ala Leu Thr Ala Asp Leu Glu Ser Leu Arg Gly
CGCCGCCAACTGCAGGCCTTGACTGCAGATCTGGAGTCCCTGCGCGGC
Gfap C291A

Fig. S4

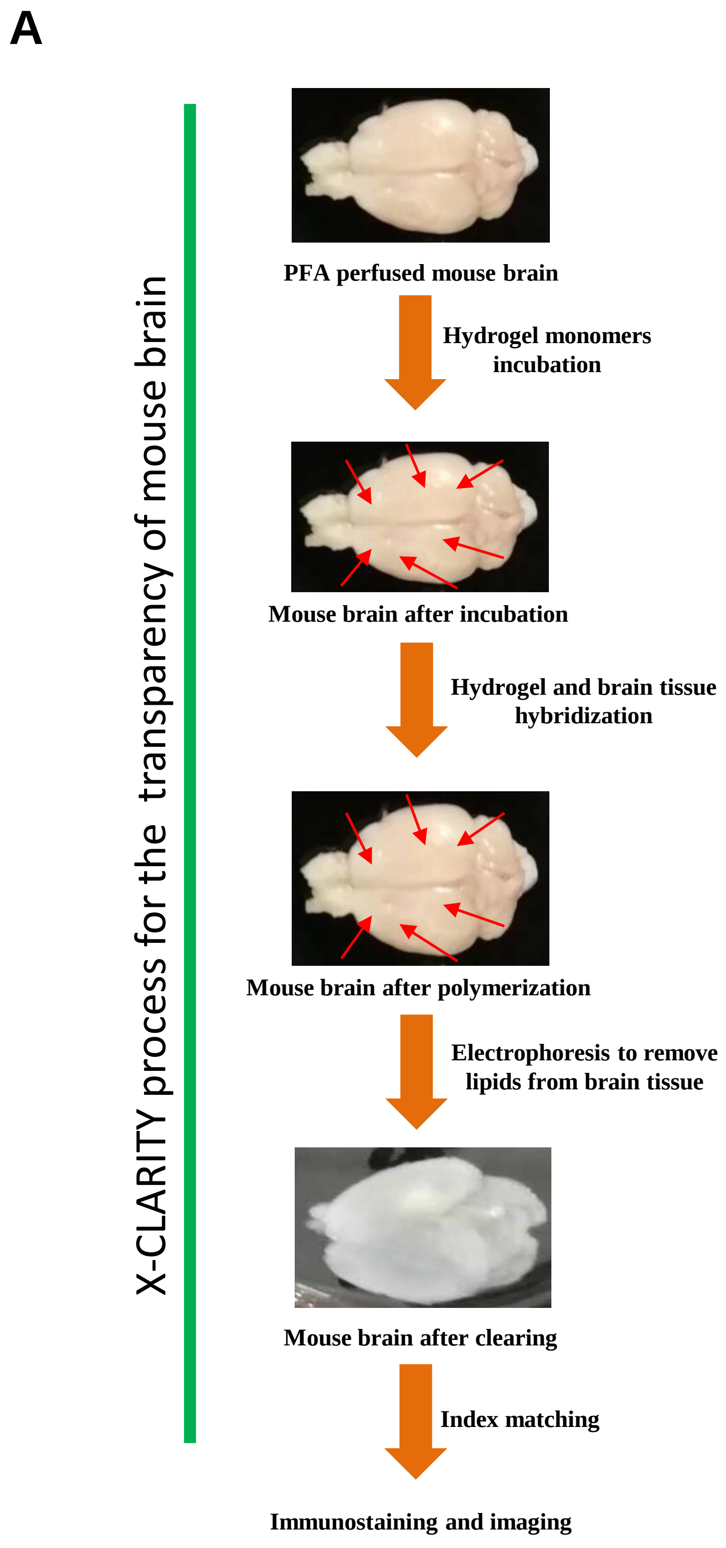
A
X-CLARITY process for the transparency of mouse brain
PFA perfused mouse brain
Hydrogel monomers incubation
Mouse brain after incubation
Hydrogel and brain tissue hybridization
Mouse brain after polymerization
Electrophoresis to remove lipids from brain tissue
Mouse brain after clearing
Index matching
Immunostaining and imaging

Fig. S5

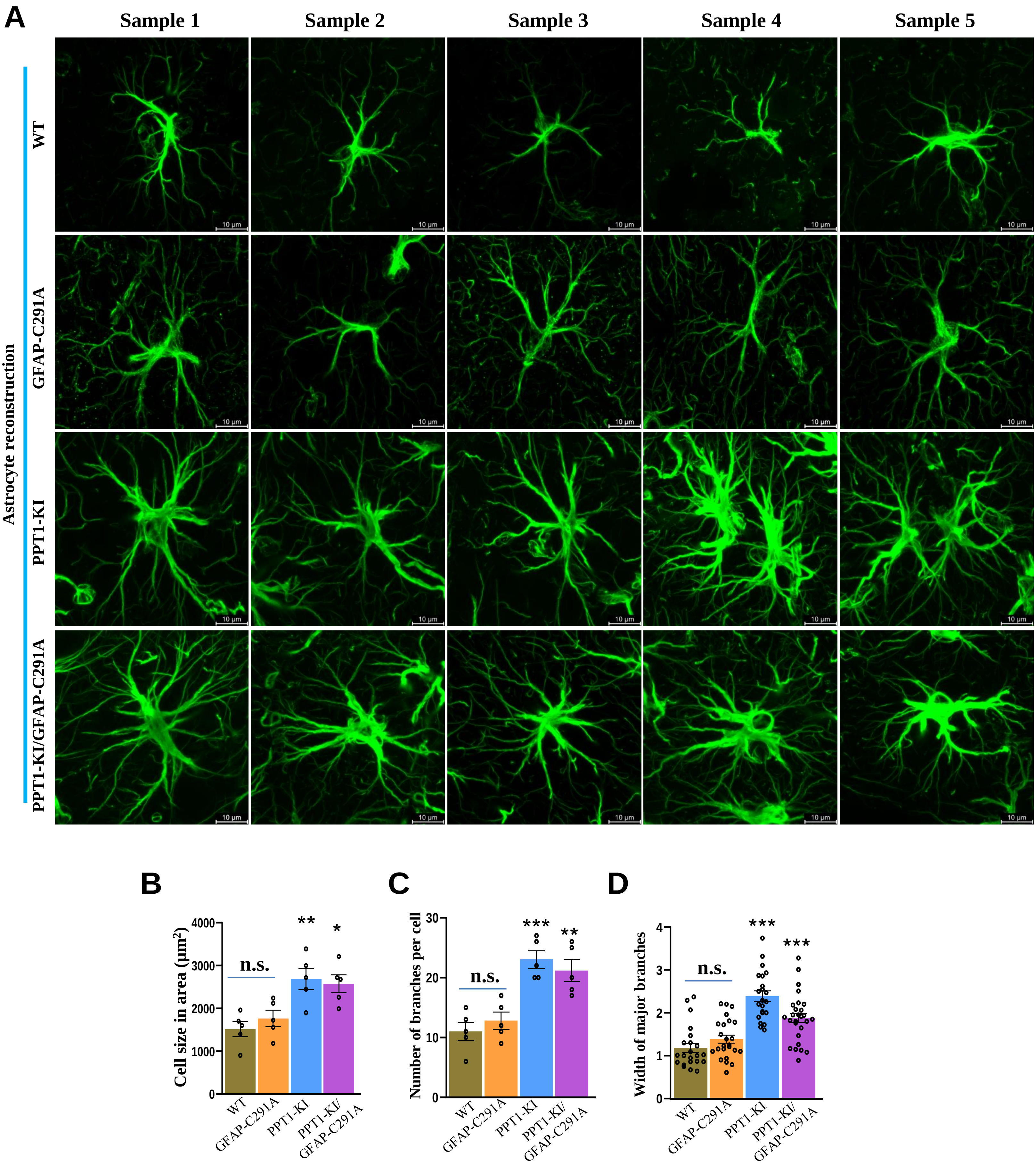
A
Sample 1
Sample 2
Sample 3
Sample 4
Sample 5
Astrocyte reconstruction
WT
GFAP-C291A
PPT1-KI
PPT1-KI/GFAP-C291A
10 μm
B
Cell size in area (μm²)
n.s.
**
*
WT
GFAP-C291A
PPT1-KI
PPT1-KI/ GFAP-C291A
C
Number of branches per cell
n.s.
***
**
D
Width of major branches
n.s.
***
***

Fig. S6

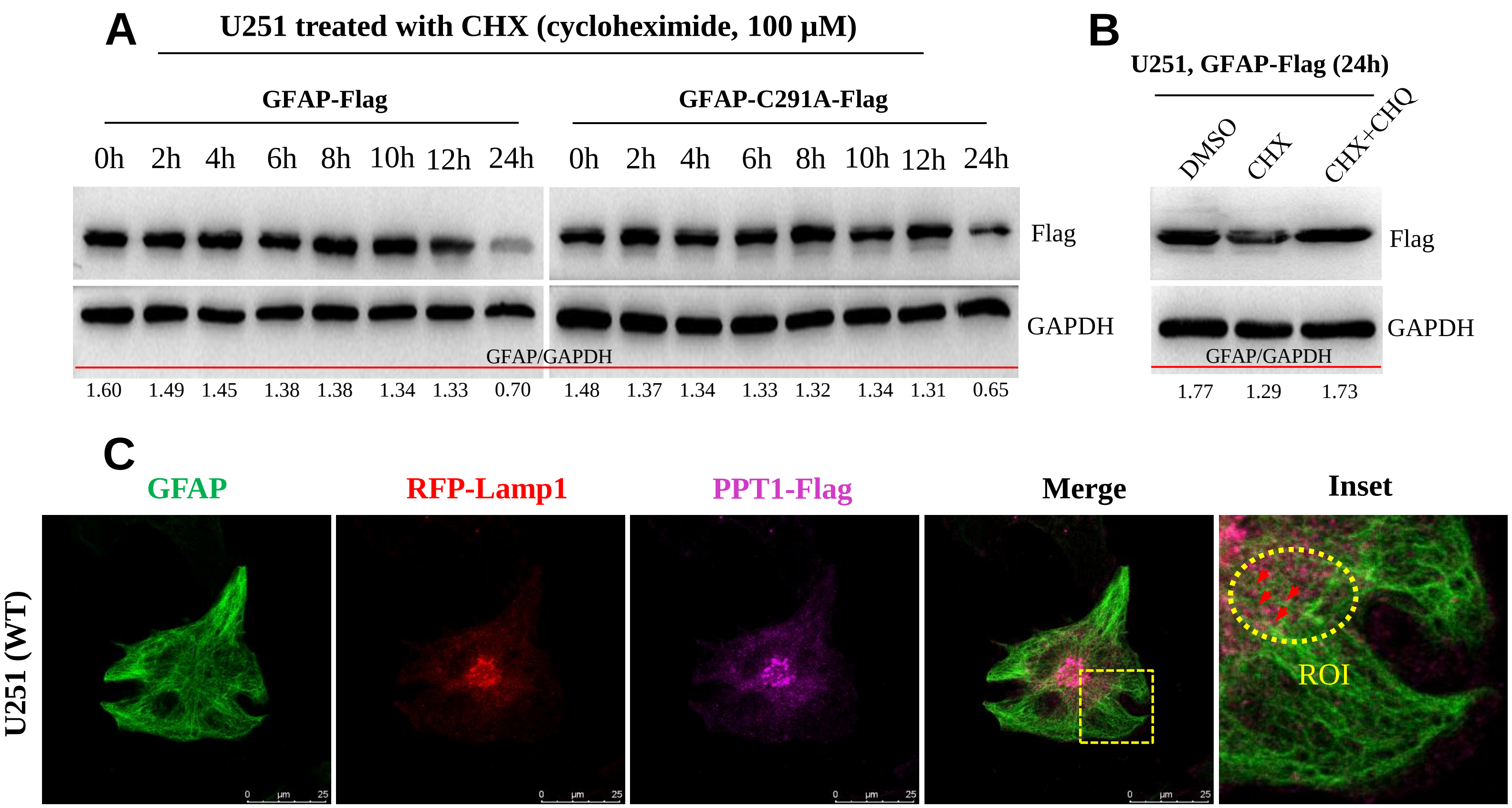

ROI	PPT1/Lamp1	PPT1/GFAP	Lamp1/GFAP
Pearson's colocalization rate	99.90%	84.78%	81.43%

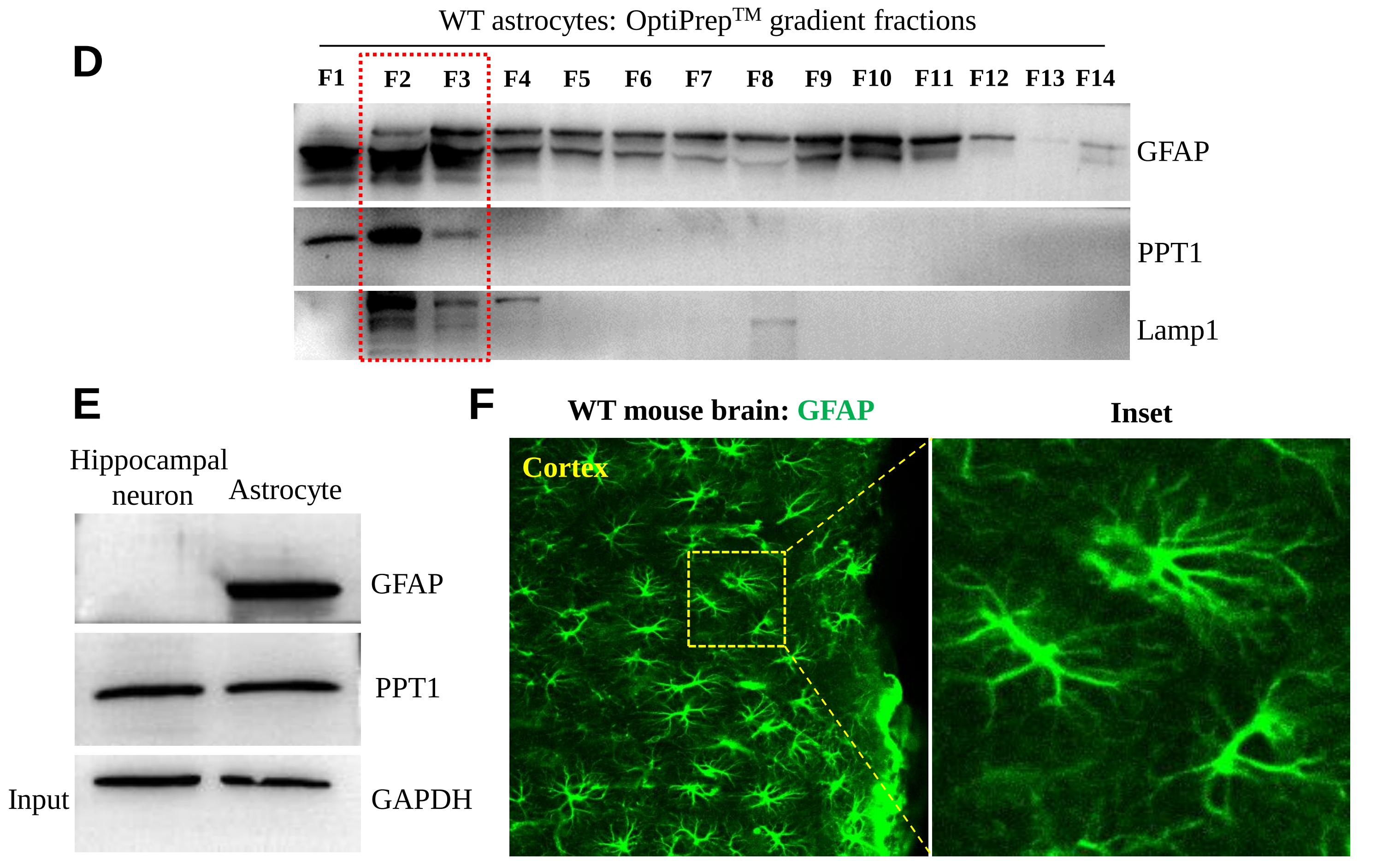

Fig. S7

Uncropped blot of Fig.1A

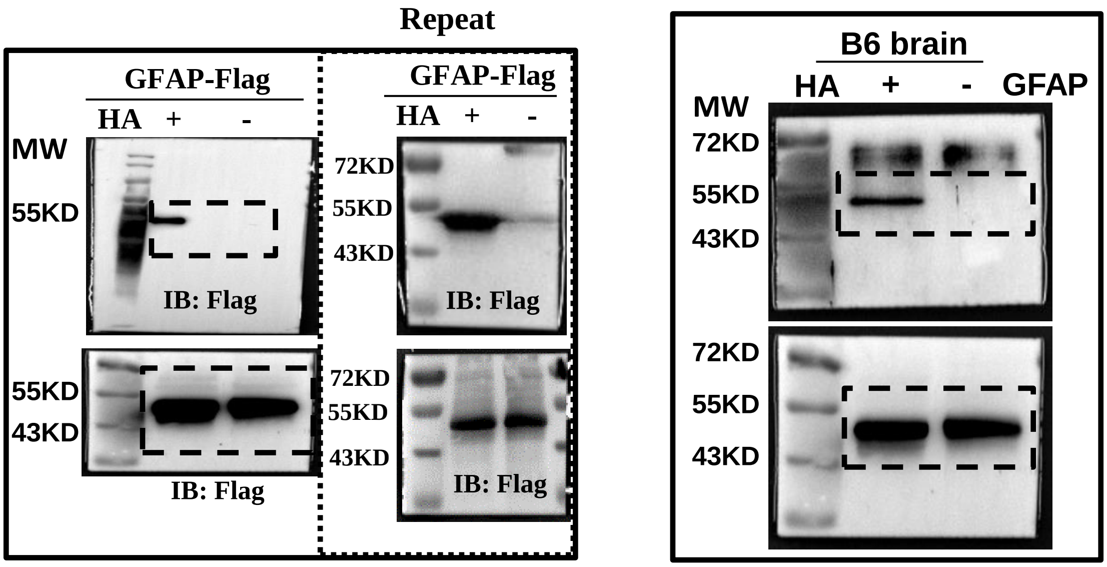

Uncropped blot of Fig.1B

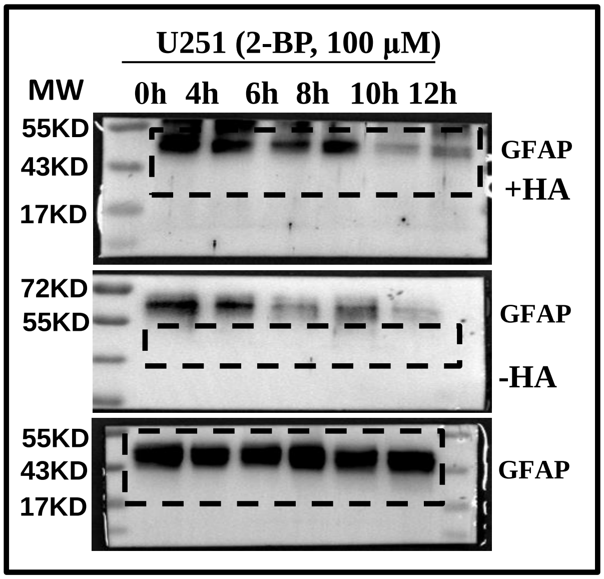

Uncropped blot of Fig.1E

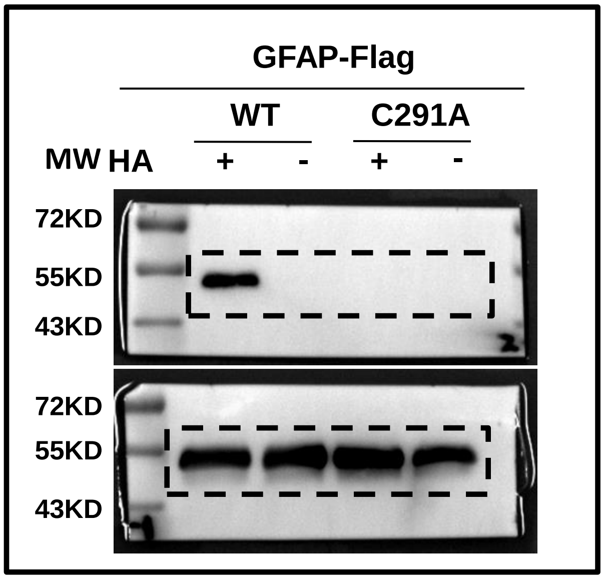

Fig. S8

Uncropped blot of Fig.3A

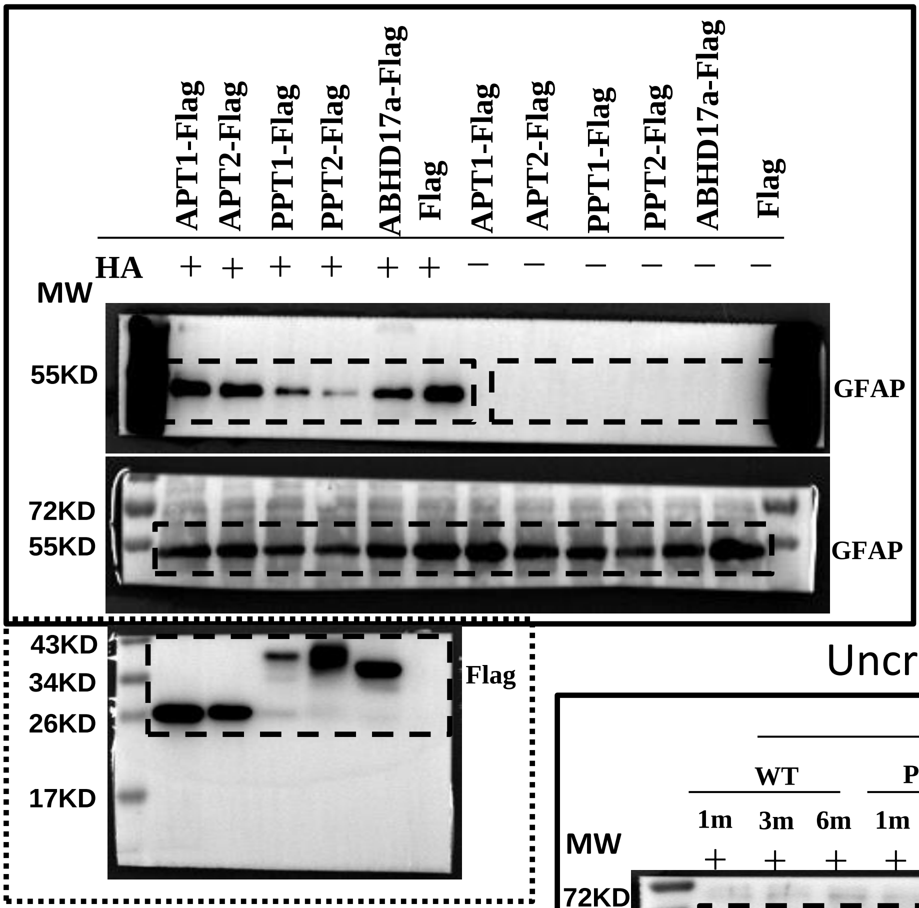

Uncropped blot of Fig.3F

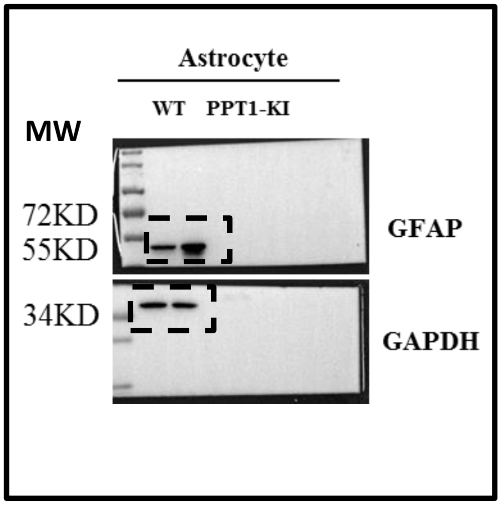

Uncropped blot of Fig.3C

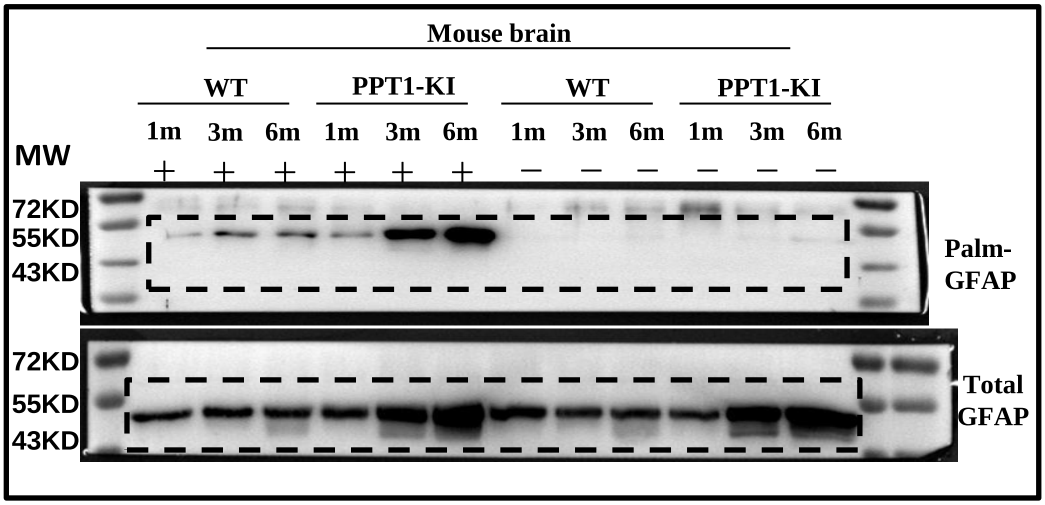

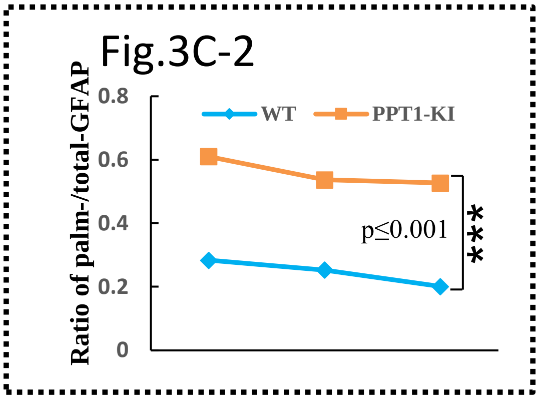

Uncropped blot of Fig.3G

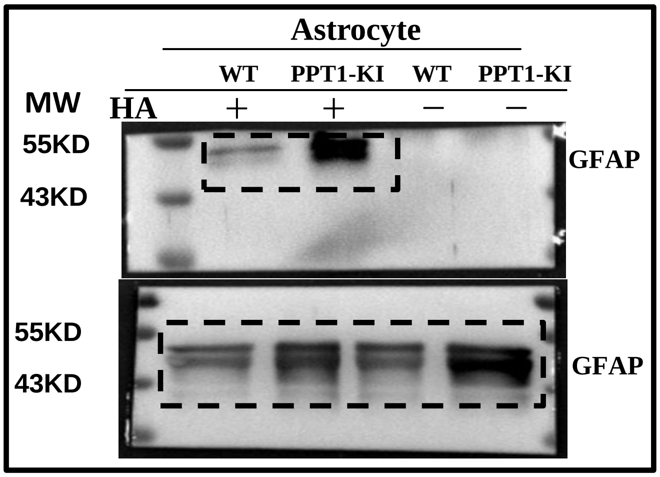

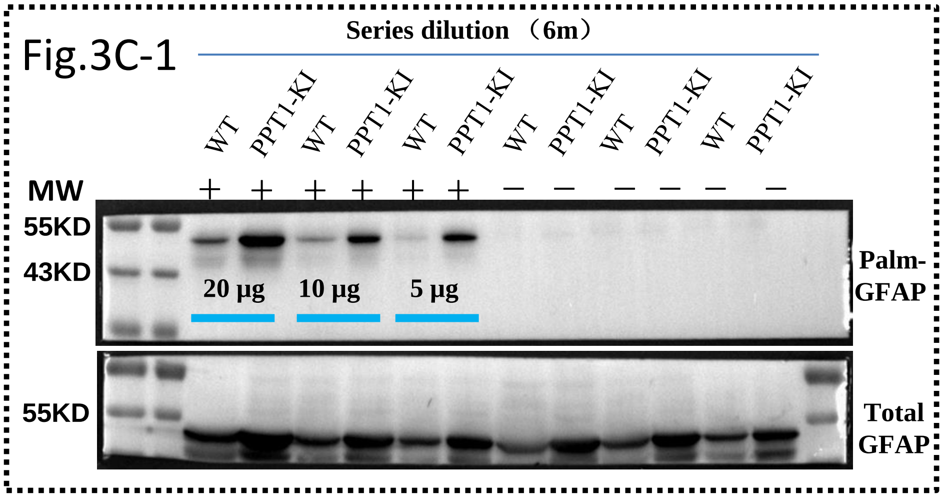

Fig. S9

Uncropped blot of Fig.4A

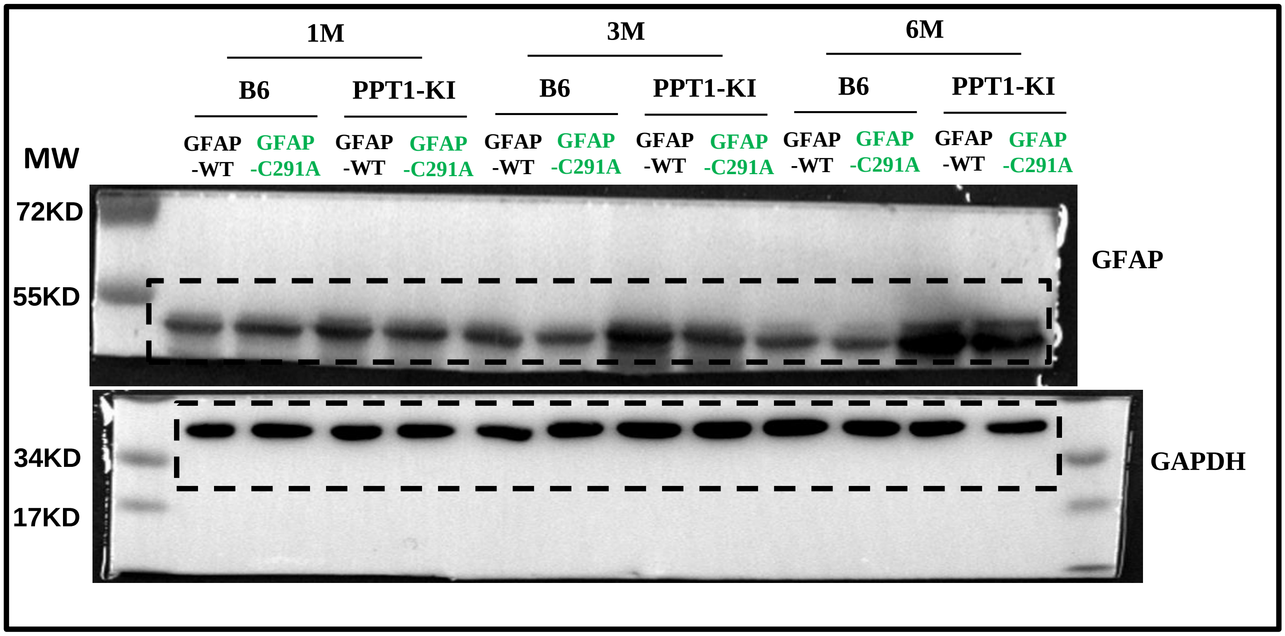

Uncropped blot of Fig.4C

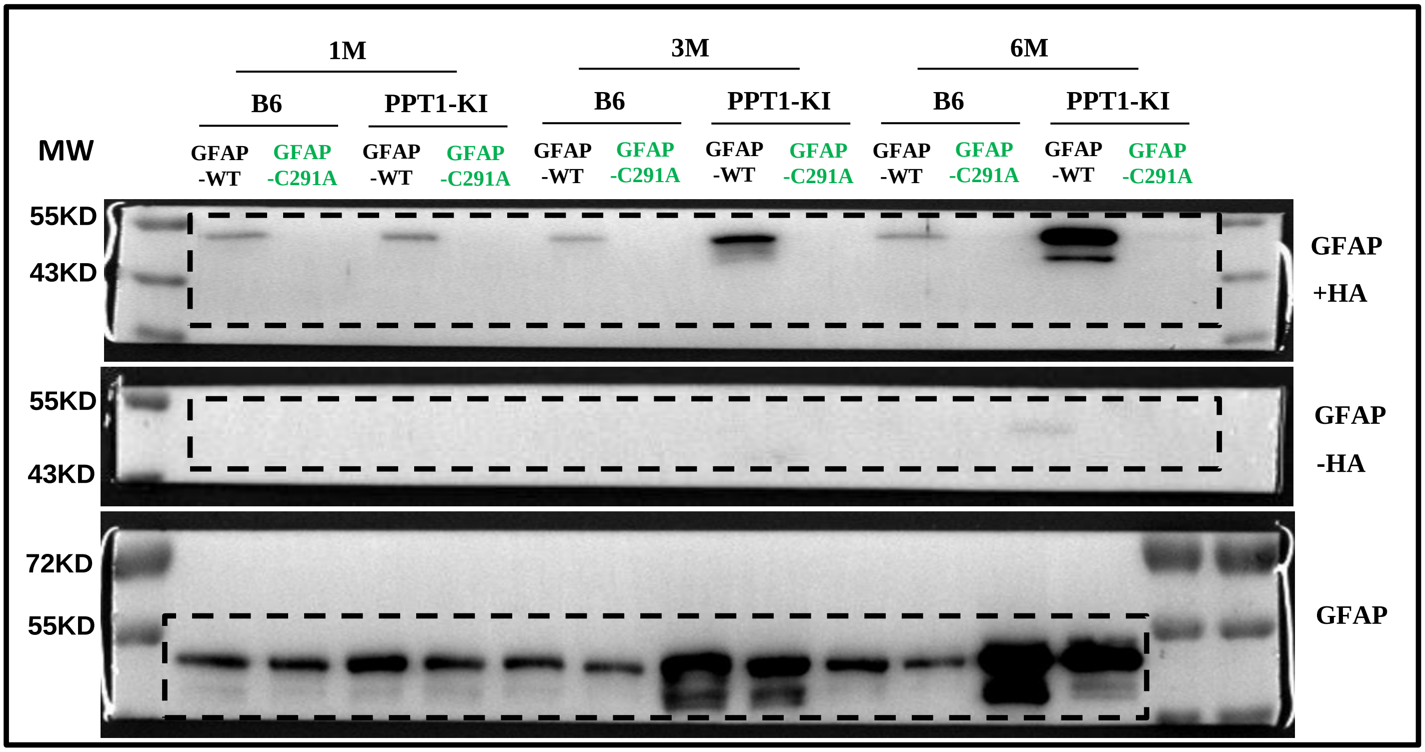

Fig. S10

Uncropped blot of Fig.5E

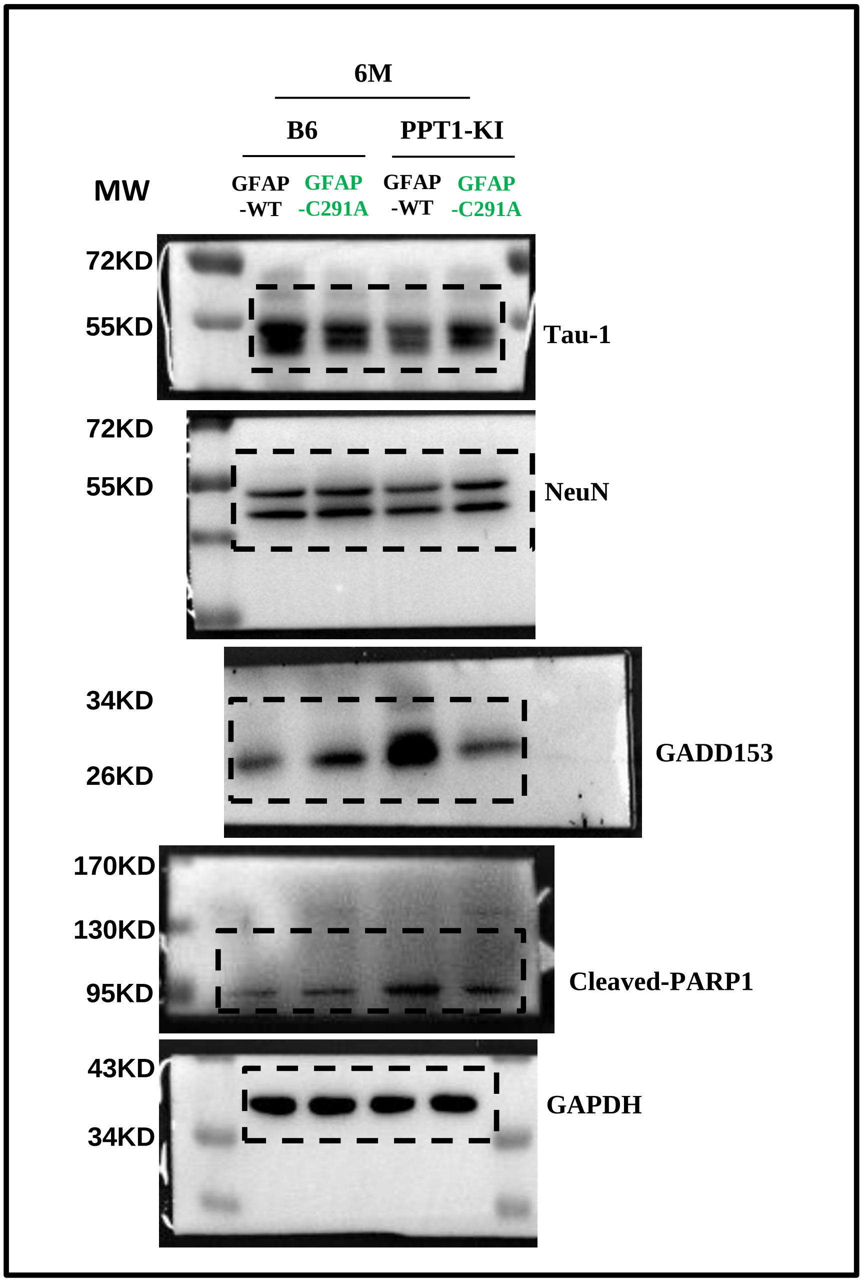

Supplement: Supplementary File [file pnas.2022261118.sapp.pdf]
